# Global geographic and socioeconomic disparities in COVID-associated acute kidney injury: a systematic review and meta-analysis

**DOI:** 10.7189/jogh.15.04166

**Published:** 2025-07-25

**Authors:** Danyang Dai, Pedro Franca Gois, Digby Simpson, Souhayel Hedfi, Sally Shrapnel, Jason Donald Pole

**Affiliations:** 1 Centre for Health Services Research, Faculty of Health, Medicine & Behavioural Sciences, The University of Queensland, Brisbane, Australia; 2 Queensland Digital Health Centre, The University of Queensland, Brisbane, Australia; 3 The University of Queensland, Brisbane, Australia; 4 Nephrology and Transplantation, John Hunter Hospital, Newcastle, Australia; 5 ARC Centre of Excellence for Engineered Quantum Systems, School of Mathematics and Physics, University of Queensland, Brisbane, Australia

## Abstract

**Background:**

Acute kidney injury (AKI) is a common and severe complication of COVID-19, which significantly increases the risk of mortality. There has been a wide range of AKI prevalence reported throughout the pandemic, reflecting differences in geographic location, patient characteristics, and health care resources. We aimed to provide a global overview of the COVID-19 AKI prevalence reported in published studies to uncover geographic and socioeconomic disparities.

**Methods:**

We undertook a systematic review and meta-analysis, searching PubMed, Embase, Scopus, Web of Science, and Cochrane Library for full-text articles published in English reporting the prevalence of AKI from January 2020 to November 2023. All studies defined AKI according to the Kidney Disease Improving Global Outcomes criteria. Clinical characteristics were extracted and examined from 334 studies that met the inclusion criteria. With significant study heterogeneity, random-effect models were estimated. We reported pooled AKI prevalence by country, region, and income level. Meta-regression further examined the relationship between COVID-associated AKI and geographic location.

**Results:**

After removing studies that utilised the same data, 345 796 patients from 246 studies were included, covering 49 countries. Of 246 studies, 137 came from high-income countries, whereas only three were conducted in low-income countries. Among non-intensive care unit (ICU) patients, low-income countries had the lowest COVID-19 AKI prevalence (14.1%; 95% confidence interval (CI) = 11.4–17.2). Among ICU patients, lower-middle-income countries had the lowest COVID-19 AKI prevalence (27.9%;95% CI = 19.4–38.4).

**Conclusions:**

Our study shows significant geographic and socioeconomic disparities in the prevalence of COVID-associated AKI, with a higher prevalence in high-income countries and a lower prevalence in low- and lower-middle-income countries. This study is the most comprehensive systematic review and meta-analysis highlighting global disparities in COVID-associated AKI prevalence. Further studies are needed to explain the reasons behind these differences.

Over the last five years, since the emergence of the severe acute respiratory syndrome coronavirus 2 (SARS-CoV-2) virus, researchers have significantly expanded our understanding of COVID-19, moving beyond respiratory symptoms to include other health complications, such as acute kidney injury (AKI) [1-4]. Many studies have shown that AKI is common among patients with COVID-19 [1-3,5]. Patients hospitalised with COVID-19 infection who develop AKI are more likely to require intensive care, invasive mechanical ventilation, experience prolonged hospital stays, and face higher mortality rates [2].

The prevalence of AKI and its impact on patient outcomes have been explored in several studies worldwide. Yet, the reported prevalence of AKI varies widely, with some studies reporting rates higher than 75% [6-8], while others report rates below 2% [9-12]. Their specific settings undoubtedly influenced the AKI prevalence reported in these studies. Systematic reviews and meta-analyses done in the early months of the pandemic [2,13-15] suggested that the reported prevalence of COVID-associated AKI was lower in studies from China and higher in the USA and Europe [5]. However, there is a lack of comprehensive systematic reviews and meta-analyses done to assess the COVID-associated AKI prevalence from a global standpoint. Assessing the true prevalence of COVID-associated AKI and understanding the substantial variation in reported prevalence across countries is essential. This knowledge may improve treatment strategies and further our understanding of the underlying disease mechanisms. We aimed to offer a global perspective on COVID-associated AKI, estimating its prevalence in different regions and evaluating the associated patient outcomes, such as mortality.

## METHODS

### Search strategy

In this systematic review and meta-analysis, we followed the PRISMA guidelines [16]. We registered the protocol in PROSPERO (registration number: CRD42023489454). Following the predetermined protocol, we systematically searched five databases, including PubMed, Embase, Scopus, Web of Science, and Cochrane Library, with assistance from a trained librarian. We used the Medical Subject Headings terms such as ‘SARS-CoV-2’, ‘COVID-19’, ‘Acute Kidney Injury’, and ‘Acute Renal Failure’ for the literature search (**Online Supplementary Document**).

### Studies selection

We considered peer-reviewed studies published from 1 January 2020 to 22 November 2023. Studies of COVID-19 hospitalised adult patients (≥18 years of age) in English were eligible for inclusion. Since we focussed on AKI prevalence, we included only those studies that used the Kidney Disease Improving Global Outcomes (KDIGO) criteria for AKI. The KDIGO is the most recent and commonly used tool for AKI [17]. The KDIGO criteria provide a standardised definition and staging system for AKI, which is recognised internationally. Limiting to studies that use the KDIGO guideline allows for comparison across different clinical settings and countries [18]. Additionally, we excluded kidney transplant studies. We considered observational, prospective, retrospective, cross-sectional, longitudinal, and cohort studies eligible study designs. We excluded case reports, reviews, clinical trials, drug test-related studies, and studies inaccessible for full-text. Three reviewers (DD, DS, and SH) independently screened titles and abstracts for all identified studies in the Covidence [19]. DD and DS, and DD and SH independently carried out full-text screening in pairs. The authors resolved discrepancies between the reviewers through collective discussion. We did not exclude studies due to small sample sizes. If a study reported identical data collected from the same hospital within a similar period, we selected studies that provided the most comprehensive information over others using the same data, to avoid overlap in the study population.

### Data extraction

Data extraction was carried out by DD and DS using a predesigned data extraction form via the Covidence system [19]. Details of the study, including study period, study population, study characteristics and the hospital where the study was conducted were extracted. Extracted study data was then randomly checked. Newcastle-Ottawa quality assessment scale (NOS) for cohort studies was used for risk of bias analysis. The NOS is widely adopted for non-randomised studies in systematic reviews [20]. The NOS assess the study's quality based on three components: selection, comparability and outcome. Stars were awarded based on the questions in each session, the maximum number of stars awarded to any study is 9. The higher the number of stars, the better the study quality according to the NOS scale [20]. Given the outcome of interest was in-hospital AKI prevalence, the question under the outcome session of the NOS was not applicable. The data extraction form and NOS questionnaire are provided in the Supplementary Materials (Table S1 in the **Online Supplementary Document**).

### Statistical analysis

The effect size of the meta-analysis was the prevalence of AKI in different geographic locations. During COVID-19, hospital admission policies varied in each country. We expected clinical heterogeneity and AKI prevalence to vary across income levels and regions [4]. We assessed the heterogeneity between studies using *I*^2^. We used a classic random effect with logit transformation using inverse-variance weights for pooling AKI prevalence. We calculated the pooled AKI prevalence and 95% confidence intervals (CIs) by different geographic regions and income levels using the ‘metaprop’ function from the meta package in *R* [21,22]. We determined the geographic areas and income levels according to the World Bank data accessed in 2024 [23]. We stratified the weighted prevalence of AKI for intensive care unit (ICU) patients and general hospitalised patients. We used a generalised linear mixed model with logit transformation for meta-regression. Logits transformation was recommended by Wolfgang Viechtbauer [24] over the Freeman-Tukey double arcsine transformation [25,26]. The Freeman-Tukey double arcsine transformation used for the proportion effect size could lead to a greater effect size than the observed proportions. While the Freeman-Tukey double arcsine transformation may provide variance-stabilising properties, it is unsuitable for meta-analysis [26]. Logit transformation offers greater interpretability and allows for a simple back-transformation, enabling clear communication of results [27]. We conducted sensitivity analyses assessing the meta-analysis results between the logits transformation and the Freeman-Tukey double arcsine transformation. We included common predictors of COVID-associated AKI, such as pre-existing chronic kidney disease (CKD) and advanced age [14], in the meta-regression analysis. We included intercepts when the meta-regression involved a continuous variable, such as mean age. For categorical variables (*e.g.* regions and income levels), we did not include an intercept. We used funnel plots and Egger's tests to assess publication biases. We conducted all statistical analysis and visualisation in R, version 4.4.2 (R Core Team, Vienna, Austria) [22].

Our secondary outcome of interest was mortality. As AKI is one of the risk factors for increased mortality among hospitalised COVID-19 patients, a higher prevalence of AKI is expected to correlate with elevated mortality rates. This association underscores the clinical significance of monitoring AKI in the context of COVID-19 outcomes. We calculated the overall pooled mortality based on the weights of each study using a random effect with logit transformation. We separately reported weighted mortality for ICU-only and general hospitalised patient studies. We carried out subgroup analysis by geographic regions and income level.

## RESULTS

### Search results

**Figure 1:**
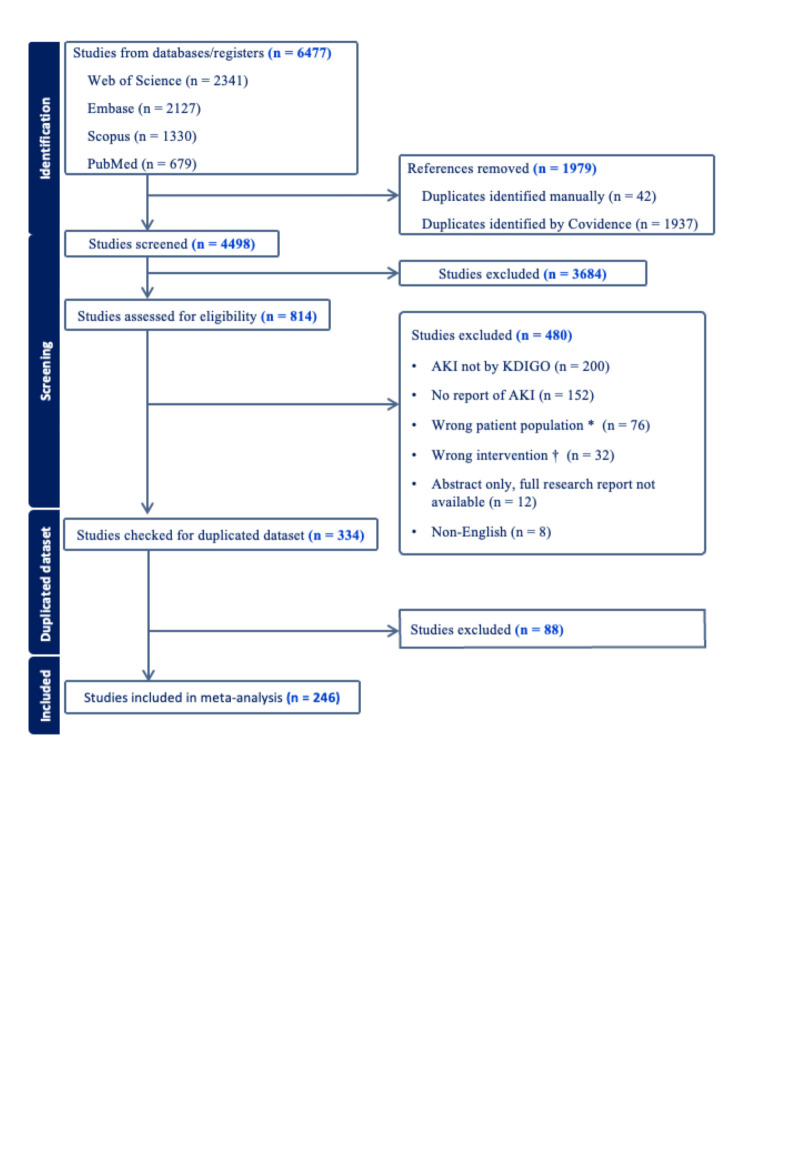
PRISMA flowchart. *Studies that included patients age <18, kidney transplanted patients, only survival patients or only death patients. †Studies that were randomised control trails, drug test related studies, studies that used propensity score matching.

We identified 6477 potentially eligible publications through the literature search. After removing duplicates, we screened 4498 studies for titles and abstracts (**Figure 1**). A total of 814 studies proceeded to full-text review. Of the 480 studies excluded, the most common reason (n = 200) was the lack of AKI diagnosis according to KDIGO criteria. After excluding duplicated cohorts, 246 studies were included in the meta-analysis. Out of 246 studies, 74 were ICU-only patients [7,8,28-81], and 46 provided separate AKI data for ICU and general ward patients [5,12,82-124]. The included studies reported 351 943 hospitalised patients from 49 countries and eight international cohorts. There were 40 studies that reported partial information for ICU patients [125-164], leading to the exclusion of 6138 patients from the meta-analysis. The study period covered from 25 December 2019 to 1 September 2022, with the final analysis including 67 853 ICU patients and 277 943 non-ICU patients.

### Study characteristics

There were eight multinational studies included in the meta-analysis [42,47,52,69,73,119,159,165], contributing 43 118 patients (Table S1 in the **Online Supplementary Document**). Among the 246 studies included in the meta-analysis, 137 reported data on COVID-19 patients from high-income countries [2,7,8,10,28,30,32,34-37,40,41,45,49-51,53,57,70,72,74-77,79-81,83,85,88-91,94,95,98,100,102,104,108,110-115,117,121,124-126,128,130-132,134-136,138,141,145,146,148,150,151,155-158,160-164,166-219], contributing 235 139 patients to the meta-analysis. The largest cohort came from the UK, which included 123 271 patients. The USA contributed 44 studies with 71 511 patients [2,50,53,62,67,76,77,83,85,95,100,112,114,130,136,146,148,151,155-157,160,162,163,170,174-178,184-187,190,198,207-209,212,214,215,218,220], followed by China with 28 studies and 32 341 patients [9,11,54,59,66,78,97,123,127,133,137,143,144,147,221-233]. In contrast, only three studies were conducted in low-income countries reporting COVID-19 [84,234,235], totalling 610 patients. By geographic regions, most studies (n = 84) were from Europe and Central Asia, encompassing 159 633 patients, while Sub-Saharan Africa had the smallest sample, with four studies and 651 patients [84,234-236]. Among 220 studies that reported age information, the reported mean age was 61.64 (standard deviation = 15.15). CKD status was reported in 118 studies, with an average prevalence of 12.90%.

### AKI prevalence

**Figure 2:**
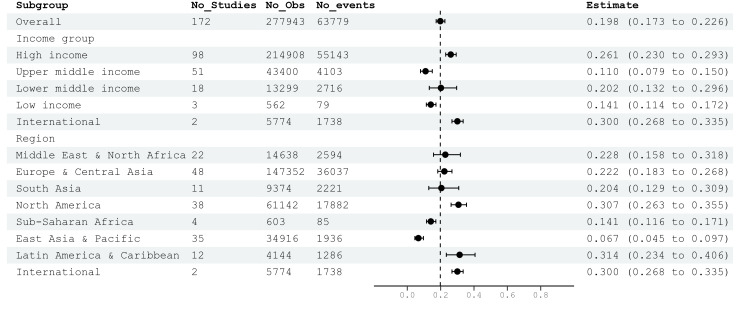
Pooled AKI prevalence for non-ICU patients by income level and region. AKI – acute kidney injury, ICU – intensive care unit.

The overall AKI prevalence was 22.95% (n = 63 779/N = 277 943) for non-ICU patients. The pooled AKI prevalence with the random effect model for non-ICU patients was 19.80% (95% CI = 17.31–22.55; n studies = 172; AKI events = 63 779; *I*^2^ = 98.9%) (Figure S1 in the **Online Supplementary Document**). By income level, the reported pooled AKI prevalence was highest for multinational studies with 30.03% (95% CI = 26.78–33.50), followed by high-income countries with 26.05% (95% CI = 23.02–29.32). There were no statistical differences in pooled AKI prevalence between multinational studies and studies conducted in high-income countries. Upper-middle-income countries had a lower reported pooled AKI prevalence with 10.96% (95% CI = 7.91–14.99) (**Figure 2**). The higher pooled AKI prevalence observed in high-income countries was influenced mainly by data from the UK and the USA, which accounted for 47 studies and included 181 167 patients (Figure S3 in the **Online Supplementary Document**). In the upper-middle income group, China was the primary contributor, with 23 studies and 30 836 patients [9,11,97,99,123,127,133,137,143,144,147,222-233]. The subgroup analysis by region suggested Latin America and Caribbean countries had a high reported pooled AKI prevalence of 31.38% (95% CI = 23.42–40.62), primarily driven by studies from Brazil and Mexico (Figure S4 in the **Online Supplementary Document**). A similar trend was observed in the ICU cohort.

**Figure 3:**
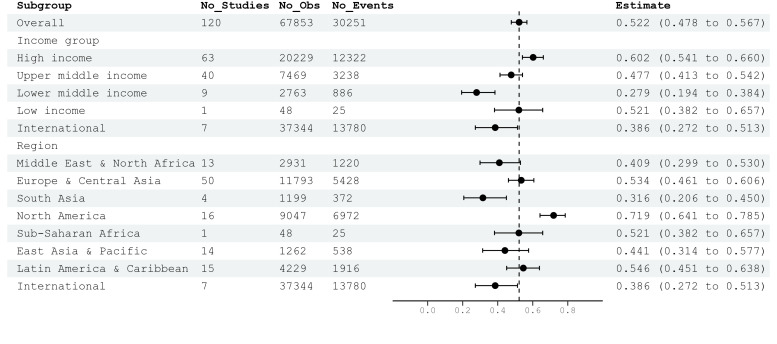
Pooled AKI prevalence for ICU patients by income level and region. AKI – acute kidney injury, ICU – intensive care unit.

Among ICU patients, AKI prevalence was 44.58% (n = 30 251/N = 67 853). Based on the random effect model, the overall AKI prevalence in ICU cohorts was 52.23% (95% CI = 47.76–56.67; n studies = 120; AKI events = 30 251; *I*^2^ = 98.4%) (Figure S2 in the **Online Supplementary Document**). Subgroup analysis by income level showed that high-income countries had higher pooled AKI prevalence (60.23%; 95% CI = 54.11–66.05) compared to upper-middle, lower-middle, and low-income countries, as well as multinational studies (**Figure 3**). Given the wide CI, there was no statistical difference in pooled COVID-associated AKI prevalence among ICU patients across different income groups. Of the 63 high-income studies included, 15 studies were from the USA (Figure S5 in the **Online Supplementary Document**). By region, North America has the highest pooled AKI prevalence (71.91%; 95% CI = 64.51–78.55) compared to other areas. Out of 16 studies from the North American region, 15 studies were from the USA and one was from Canada (Figure S6 in the **Online Supplementary Document**)

We carried out a meta-regression to further investigate the relationship between AKI prevalence and geographic locations (**Table 1**). The omnibus tests for each model of ICU patients indicated that the jointed statistics among moderators were insignificant. This suggests that region and income level were not significantly associated with AKI prevalence among ICU patients. For non-ICU patients, regions, and income levels were found to be correlated with AKI prevalence. The mean age of patients and pre-existing CKD condition were shown to be a risk factor contributing to AKI for non-ICU patients when evaluated independently. For non-ICU COVID-19 patients, each additional year of age increases the risk of developing AKI by 5.4%. There were 76 studies that reported both mean age and CKD percentage for non-ICU patients, with no studies from Sub-Saharan Africa. Controlling for both mean age and pre-existing CKD, the non-ICU patient model indicates that region is strongly correlated with AKI prevalence. After accounting for pre-existing CKD and the mean age for each study population, published studies from South Asia countries reported a 6.98-fold higher AKI prevalence in non-ICU patients compared to East Asia and Pacific countries. The income level factor indicates that international studies reported the highest AKI prevalence. However, meta-regression on ICU patients revealed no significant factors contributing to the high AKI prevalence.

**Table 1:** Meta-regression stratified by ICU status

	Non-ICU																	
	**Regions**			**Age**			**Region + age**			**CKD**			**Region + CKD**			**Region + CKD + age**		
	***β* (SE)**	***P*-value**	**OR**	***β* (SE)**	***P*-value**	**OR**	***β* (SE)**	***P*-value**	**OR**	***β* (SE)**	***P*-value**	**OR**	***β* (SE)**	***P*-value**	**OR**	***β* (SE)**	***P*-value**	**OR**
**East Asia & Pacific**	−2.610 (0.161)	<0.0001	0.074				ref						ref			ref		
**Europe & Central Asia**	−1.254 (0.133)	<0.0001	0.285				0.873 (0.236)	<0.0001	2.394				1.299 (0.257)	<0.0001	3.664	1.062 (0.312)	0.0007	2.891
**International**	−0.846 (0.637)	0.1841	0.429				1.478 (0.629)	0.0002	4.383				1.606 (0.550)	0.0035	4.981	1.482 (0.563)	0.0085	4.401
**Latin America & Caribbean**	−0.783 (0.265)	0.0031	0.457				1.684 (0.312)	0.0189	5.389				1.428 (0.357)	<0.0001	4.168	1.292 (0.398)	0.0011	3.637
**Middle East & North Africa**	−1.220 (0.195)	<0.0001	0.295				1.491 (0.269)	<0.0001	4.441				1.532 (0.311)	<0.0001	4.628	1.441 (0.324)	<0.0001	4.225
**North America**	−0.812 (0.148)	<0.0001	0.444				1.396 (0.243)	<0.0001	4.04				1.771 (0.252)	<0.0001	5.877	1.518 (0.296)	<0.0001	4.562
**South Asia**	−1.358 (0.275)	<0.0001	0.257				1.264 (0.333)	0.0001	3.54				1.537 (0.464)	0.0009	4.649	1.944 (0.561)	0.0005	6.984
**Sub-Saharan Africa**	−1.819 (0.475)	0.0001	0.162				0.987 (0.553)	0.0744	2.683									
**Intercept**				−4.573 (0.664)	<0.0001	0.01	−4.996 (0.652)	<0.0001	0.007	−1.606 (0.145)	<0.0001	0.201	−2.604 (0.181)	<0.0001	0.074	−3.836 (0.795)	<0.0001	0.022
**Age**				0.052 (0.011)	<0.0001	1.054	0.043 (0.011)		1.044							0.022 (0.014)	0.1021	1.022
**CKD**										2.849 (0.796)	0.0003	17.265	1.316 (0.709)		3.729	1.172 (0.728)	0.1074	3.227
**Number of studies**	172			145			145			86			86			76		
**Test of moderators, *P*-value**	<0.0001			<0.0001			<0.0001			0.0003			<0.0001			<0.0001		
***I*^2^, %**	99.4			99.33			99			99.47			98.9			98.5		
**R^2^**				0.1344			0.361			0.1251			0.461			0.458		
	**ICU**																	
**East Asia & Pacific**	−0.236 (0.261)	0.3643	0.79				ref						ref			ref		
**Europe & Central Asia**	0.129 (0.131)	0.3244	1.138				0.464 (0.333)	0.1629	1.591				0.330 (0.462)	0.4751	1.391	0.372 (0.494)	0.4507	1.451
**International**	−0.465 (0.340)	0.1715	0.628				−0.134 (0.501)	0.7888	0.874				−0.279 (0.570)	0.6244	0.756	−0.132 (0.626)	0.8335	0.877
**Latin America & Caribbean**	0.190 (0.238)	0.4236	1.209				0.503 (0.385)	0.1908	1.654				0.274 (0.519)	0.5984	1.315	0.388 (0.536)	0.4696	1.473
**Middle East & North Africa**	−0.366 (0.254)	0.1496	0.693				0.223 (0.415)	0.5912	1.249				0.335 (0.655)	0.6091	1.398	0.504 (0.671)	0.4525	1.655
**North America**	0.936 (0.226)	<0.0001	2.55				1.210 (0.389)	0.0019	3.354				0.966 (0.536)	0.0715	2.629	1.037 (0.557)	0.0629	2.82
**South Asia**	−0.775 (0.452)	0.0863	0.461				−0.366 (0.543)	0.5003	0.693									
**Sub-Saharan Africa**	0.083 (0.938)	0.9292	1.087				0.530 (0.994)	0.5935	1.7									
**Intercept**				−1.691 (1.008)	0.0934	0.184	−1.158 (1.022)	0.2568	0.314	−0.017 (0.233)	0.9403	0.983	−0.175 (0.383)	0.6481	0.839	−2.331 (1.585)	0.1414	0.097
**Age**				0.029 (0.016)	0.0721	1.03	0.014 (0.017)	0.4198	1.014							0.035 (0.026)	0.1869	1.035
**CKD**										1.959 (1.589)	0.2176	7.092	0.419 (1.780)	0.8137	1.521	−0.293 (1.872)	0.8754	0.746
**Number of studies**	120			104			104			56			56			53		
**Test of moderators, *P*-value**	0.0009			0.0721			0.0083			0.2176			0.2043			0.1888		
***I*^2^, %**	98.21			98.28			97.85			99.07			98.73			98.14		
**R^2^**				0.0264			0.1252			0.0065			0.0516			0.0527		
	**Non-ICU**																	
	**Income**			**Age**			**Income + age**			**CKD**			**Income + CKD**			**Income + age + CKD**		
	***β* (SE)**	***P*-value**	**OR**	***β* (SE)**	***P*-value**	**OR**	***β* (SE)**	***P*-value**	**OR**	***β* (SE)**	***P*-value**	**OR**	***β* (SE)**	***P*-value**	**OR**	***β* (SE)**	***P*-value**	**OR**
**High income**	−1.049 (0.102)	<0.0001	0.35				ref						ref			ref		
**International**	−0.846 (0.703)	0.2284	0.429				0.343 (0.693)	0.6205	1.409				0.194 (0.604)	0.7484	1.214	0.317 (0.595)	0.5937	1.374
**Low income**	−1.835 (0.591)	0.0019	0.16				−0.296 (0.722)	0.6816	0.744									
**Lower middle income**	−1.375 (0.238)	<0.0001	0.253				0.275 (0.304)	0.3661	1.316				0.352 (0.367)	0.337	1.423	0.742 (0.398)	0.0623	2.1
**Upper middle income**	−2.075 (0.143)	<0.0001	0.126				−0.615 (0.215)	0.0042	0.541				0.989 (0.232)	<0.0001	0.372	−0.770 (0.263)	0.0034	0.463
**Intercept**				−4.573 (0.664)	<0.0001	0.01	−3.621 (0.816)	<0.0001	0.027	−1.606 (0.145)	<0.0001	0.201	−1.239 (0.169)	<0.0001	0.29	−3.099 (0.922)	0.0008	0.045
**Age**				0.052 (0.011)	<0.0001	1.05	0.039 (0.012)	0.0014	1.04							0.029 (0.014)	0.0404	1.029
**CKD**										2.849 (0.796)	0.0003	17.265	1.716 (0.774)	0.0265	5.562	1.392 (0.769)	0.0701	
**Number of studies**	172			145			145			86			86			76		
**Test of moderators, *P*-value**	<0.0001			<0.0001			<0.0001			0.0003			<0.0001			<0.0001		
***I*^2^, %**	99.55			99.33			99.26			99.47			99.31			98.88		
**R^2^**				0.1344			0.1861			0.1251			0.2902			0.3259		
	**ICU**																	
**High income**	0.415 (0.116)	0.0003	1.514				ref						ref			ref		
																		
**International**	−0.465 (0.338)	0.1692	0.628				−0.832 (0.428)	0.0516	0.435				−0.782 (0.445)	0.079	0.457	−0.736 (0.494)	0.1361	0.479
**Low income**	0.083 (0.934)	0.9288	1.087				−0.121 (0.974)	0.9014	0.886									
**Lower middle income**	−0.953 (0.301)	0.0015	0.386				−0.985 (0.374)†	0.0085	0.374				−0.409 (0.935)	0.6614	0.664	−0.450 (0.941)	0.6325	0.638
**Upper middle income**	−0.087 (0.147)	0.555	0.917				−0.470 (0.208)	0.0237	0.625				−0.291 (0.300)	0.3312	0.747	−0.375 (0.307)	0.2213	0.687
**Intercept**				−1.691 (1.008)	0.0934	0.184	−0.815 (1.019)	0.4238	0.443	−0.017 (0.233)	0.9403	0.983	0.232 (0.291)	0.4247	1.261	−1.967 (1.549)	0.2042	0.14
**Age**				0.029 (0.0163)	0.0721	1.03	0.019 (0.016)	0.2327	1.02							0.037 (0.026)	0.1436	1.038
**CKD**										1.959 (1.589)	0.2176	7.092	1.291 (1.669)	0.4392	3.637	0.350 (1.811)	0.8469	1.418
**Number of studies**	120			104			104			56			56			53		
**Test of moderators, *P*-value**	0			0.072			0.008			0.218			0.286			0.234		
***I*^2^, %**	98.2			98.3			97.9			99.1			98.8			98.2		
**R^2^**				0.026			0.107			0.007			0.024			0.034		

CKD – chronic kidney disease, ICU – intensive care unit, OR – odds ratio, ref – reference, SE – standard error, *β* – correlation coefficient

### Mortality

There were 215 studies reporting mortality (**Table 2**). For ICU-only studies, the overall pooled mortality was 31.03% (95% CI = 26.56–35.89) over 70 studies [7,8,29-42,44-66,68-74,76-81,165-167,169-172,194,199,221,237-244]. For studies that included patients hospitalised with COVID-19, where mortality data could not be separately extracted for ICU and non-ICU patients, the pooled mortality was 17.95% (95% CI = 15.67–20.48) over 145 studies [2,9,11,82-84,86-98,100-103,105-108,110-117,119-123,125-141,143-145,147-156,158-163,173,175-177,179,180,182,183,185,186,188-193,195-202,204-207,209-211,213-217,219,223-226,228,229,231-234,236,245-259]. Most of the studies reporting COVID-19 mortality were from high-income countries (54.88%) followed by upper-middle income countries (31.63%). By regions, the Middle East and North Africa reported the highest pooled mortality (41.84%; 95% CI = 31.62–52.82) among ICU-only studies. In studies that included patients hospitalised with COVID-19, Latin America and Caribbean countries reported a high pooled mortality (35.62%; 95% CI = 23.10–50.47). In contrast, East Asia and the Pacific studies reported the lowest mortality for hospitalised patients (7.98%; 95% CI = 4.89–12.76) and ICU-only studies (16.48%; 95% CI = 5.45–40.32).

**Table 2:** Meta-analysis for pooled mortality

	General hospital studies				
	**Random effect (95% CI)**	**Number of studies**	**Number of observations**	**Number of deaths**	***I*^2^, %**
**Overall**	0.180 (0.1567–0.2048)	145	300 476	54 170	98.90
**Region**					
East Asia & Pacific	0.080 (0.0489–0.1276)	30	33 539	2875	97.90
Europe & Central Asia	0.202 (0.1761–0.2309)	45	107 925	30 943	97.40
International	0.218 (0.1675–0.2794)	2	11 373	2431	98.10
Latin America & Caribbean	0.356 (0.2310–0.5047)	9	2980	1017	97.80
Middle East & North Africa	0.185 (0.1515–0.2227)	19	13 463	2434	93.80
North America	0.268 (0.2190–0.3244)	27	51 494	12 653	99.30
South Asia	0.138 (0.0783–0.2316)	10	9728	1733	97.60
Sub-Saharan Africa	0.172 (0.0794–0.3337)	3	549	84	92.40
**Income level**					
High	0.215 (0.1902–0.2422)	82	165 361	44 468	98.70
Upper middle	0.129 (0.0887–0.1839)	45	42 536	5065	98.50
Lower middle	0.143 (0.0888–0.2219)	14	11 273	2127	97.10
Low	0.195 (0.0623–0.4692)	2	508	79	96.10
International	0.218 (0.1675–0.2794)	2	11 373	2431	98.10
	**ICU-only studies**				
**Overall**	0.310 (0.2656–0.3589)	70	51 458	18 203	96.90
**Region**					
East Asia & Pacific	0.165 (0.0545–0.4032)	7	931	272	94.80
Europe & Central Asia	0.340 (0.2789–0.4075)	34	9692	3299	96.00
International	0.251 (0.1649–0.3617)	6	33 269	12 385	98.40
Latin America & Caribbean	0.260 (0.1792–0.3615)	10	3061	649	94.50
Middle East & North Africa	0.418 (0.3162–0.5282)	5	1780	752	94.40
North America	0.326 (0.2225–0.4503)	6	1204	392	94.90
South Asia	0.335 (0.0064–0.9751)	2	718	454	99.30
**Income level**					
High	0.314 (0.2637–0.3699)	36	10 536	3506	95.50
Upper middle	0.310 (0.2202–0.4165)	23	4978	1409	96.60
Lower middle	0.357 (0.1210–0.6904)	5	1872	903	98.80
International	0.251 (0.1649–0.3617)	6	33 269	12 385	98.40

CI – confidence interval, ICU – intensive care unit

### Sensitivity analysis

Using the Freeman-Tukey double arcsine transformation and a random-effects model, the overall pooled AKI prevalence for non-ICU patients was 22.01% (95% CI = 19.59–24.52; n studies = 172; n patients = 277 943; AKI events = 63 779; *I*^2^ = 99.3%). The pooled AKI prevalence for ICU patients was 52.11% (95% CI = 47.92–56.28; n studies = 120; n patients = 67 853; AKI events = 30 251; *I*^2^ = 98.9%). Given the results using Freeman-Tukey double arcsine transformation and logit transformation were similar, the results appear robust.

### Risk of bias and study quality

Most studies (92.27%) scored six out of nine and above on the Newcastle-Ottawa Scale quality assessment scale (Table S1 in the **Online Supplementary Document**). Some studies did not report mortality or CKD. Given this, these studies received no stars in the comparability section of the Newcastle-Ottawa Scale cohort study scale as they did not satisfy the comparability of cohorts based on the design or analysis. As all studies were conducted using hospital records, each study received one point to ascertain exposure under the selection criteria. Over 85% of the studies used polymerase chain reaction or rapid antigen self-tests for their COVID-19 diagnoses. Out of 246 studies, 187 started and were completed within 2020, and 38 started in 2020 and were completed in 2021. Only 11 studies were conducted after 2021.

### Publication bias

Analysis using funnel plots and Egger's tests for the prevalence of AKI suggested some potential bias (Figures S7 and S8 in the **Online Supplementary Document**). The results indicated that studies with smaller sample sizes tended to report marginally lower AKI prevalence.

## DISCUSSION

To our knowledge, our study is the largest and most comprehensive systematic review and meta-analysis focussing on COVID-associated AKI among hospitalised patients worldwide. We included 345 796 patients hospitalised with COVID-19 from 246 cohort studies across 49 countries and eight international studies covering non-ICU and ICU populations. The mean AKI prevalence for non-ICU hospitalised patients was 22.95%, but ranged from 0 in Taiwan, China [10] to 64.57% in New York, USA [215]. For ICU patients, the AKI prevalence was 44.58% and ranged from 11.76% in London, UK [194] to 95.12% in Rozzano, Italy [75]. There were 215 studies that reported mortality. The overall pooled mortality for non-ICU was 17.95% and 31.03% for ICU mortality.

A key finding of our study is the significant geographic variation in the prevalence of COVID-associated AKI, a pattern that underscores global disparities and adds new insights to the existing body of research [2,4]. Previous meta-analysis addressing COVID-associated AKI suggested that the pooled incidence of AKI from Asian countries was 5.5%, whereas studies from the USA and Europe reported a pooled AKI incidence of 28.6% [4]. Another systematic review and meta-analysis with subgroup analysis comparing Asian vs non-Asian AKI suggested a similar pattern, albeit with a lower incidence overall [260]. They found the pooled AKI incidence was 7% for the Asian group, where 15% of AKI incidence was found in the non-Asian group [260]. Our meta-regression analysis showed that geographic location affects the occurrence of COVID-associated AKI in non-ICU patients. Studies have found that COVID-19 patients who developed AKI had a higher risk of in-hospital death [5,15,73,90]. This aligns with the findings from our meta-analysis. We observed a positive correlation between AKI prevalence and mortality, with the Latin America and Caribbean region reporting both the highest AKI prevalence and mortality among non-ICU patients.

Mortality rates among non-ICU patients varied across geographic regions and income groups, which may reflect variations in hospital admission policies during the pandemic [4]. However, other contextual factors and potential confounders could also have contributed to these differences. Early in the pandemic, China implemented several unique hospitalisation policies compared to other countries. For example, the guideline recommendation regarding hospital admission in China by the National Health Commission specifies that all suspected and confirmed cases must be treated in designated hospitals with adequate isolation and protective measures [261]. With limited hospital beds, China converted public areas such as conference centres and stadiums into Fangcang shelter hospitals [262,263]. Thus, patients with mild to moderate signs or symptoms were admitted to Fangcang Shelter Hospitals [264]. Compared to China’s authoritarian governance towards ‘zeroing’ and ‘dynamic zeroing’ policy [265,266], the US ultimately adopted the ‘coexistence’ policy with a limited political system in place [265]. The US Centers for Disease Control and Prevention emphasised prioritising patients with severe symptoms or those at high risk of complications [267]. This is supported by another systematic review, which found that North American patients with COVID-19 were more likely to be aged ≥65 and had a higher incidence of AKI compared to their Asian counterparts [268]. Our data also found that COVID-19 patients admitted to hospitals in the US tended to be older, and more patients had CKD. The differences in COVID-19 management policy might be contributing to the variation of AKI prevalence among non-ICU patients across the world, where there were no differences in AKI prevalence among ICU patients. This suggests that the ICU admission was consistent globally, where the hospital admission policy differs among different geographical locations. Attributing these differences solely to disparities in health care resources or hospital admission policies may be an oversimplification as other factors and potential confounders could impact these results. Other than COVID-19 policy differences, historical records of AKI prevalence also suggested that the Eastern Asian region tended to have a lower AKI prevalence compared to other areas [269].

We also investigated other risk factors, beyond geographic location, for COVID-associated AKI. In our analysis, we identified that higher age and pre-existing CKD contributed to the development of COVID-associated AKI, as previously published [90,268,270]. One meta-analysis found that a one-year increment would increase the risk of COVID-associated AKI by 2%, and pre-existing CKD had an odds ratio of 2.55 [270]. Advanced age and pre-existing CKD are well-established risk factors for AKI [90,268,270]. Zhang and colleagues demonstrated that each additional year of age increases the risk of COVID-associated AKI by 2%, while pre-existing CKD raises the odds of developing AKI by 2.55 times [270]. In our study, we observed similar trends, with age and pre-existing CKD contributing to the development of COVID-associated AKI. We also included these factors as confounders in the meta-regression for regional and socioeconomic analyses. After adjusting for age and pre-existing CKD, we found that South Asia had the highest risk of COVID-associated AKI among non-ICU patients. This may also help explain the higher AKI prevalence observed in North America, where hospitalised patients were older and had more comorbidities [268].

By income level, a meta-analysis also identified a lower AKI incidence among lower-middle (13.5%) and upper-middle (19.5%) countries compared to high-income (23.8%) countries [269]. Similar trends were observed in our systematic review, with high-income countries reporting higher pooled AKI prevalence. However, the difference in COVID-associated AKI prevalence was not found to be statistically different in our systematic review. Cohort studies from the pre-pandemic era hypothesised that diagnostic bias might partially explain the lower reported AKI prevalence in lower-income countries, as these nations allocate less funding to health care and laboratory testing compared to higher-income countries [269]. These disproportionate representations of certain countries highlight a significant gap in the existing AKI literature. Establishing multinational, interdisciplinary AKI networks offers a potential solution to address this disparity and bridge the gap between lower-income and high-income countries in AKI research.

Our study has several limitations. First, due to kidney replacement therapy being reported in fewer than 50% of the studies, we could not incorporate it into our analysis. Second, more than 85% of the studies included in this analysis came from high-income and upper-middle-income countries, resulting in under-representation of lower-middle- and low-income countries. This imbalance may be attributed to better access to laboratory testing in higher-income countries and publication bias [42]. Third, as a systematic review and meta-analysis based on published studies, the lack of studies from low- and lower-middle-income countries may explain the differences between meta-analysis findings and observational studies with individual patients’ data [42]. Fourth, the heterogeneity among the included studies was high, with *I*^2^ exceeding 95% in most analyses [271]. Fifth, although some may question the use of geographic meta-regression comparisons, it remains a widely adopted and, to some extent, unavoidable method when reviewing studies from multiple countries [268,272]. Lastly, given that variations in AKI diagnostic criteria would lead to different AKI incidence, we only included studies that explicitly applied the KDIGO AKI definition [273]. This approach enhances the methodological consistency and ensures the robustness and comparability of the pooled results across countries. These strict inclusion criteria may limit the potential generalisability of these results. Given this is a systematic review, we had to rely on the authors of each study to apply the KDIGO criteria appropriately. This systematic review and meta-analysis represent the most extensive examination of COVID-associated AKI, incorporating data from many countries and diverse populations. Our study's strengths lie in the large number of studies analysed, which enables a more precise prevalence estimation and the ability to perform subgroup analysis across different socioeconomic levels and geographic regions. However, the paucity of data from lower-middle and low-income countries highlights the need for further research on COVID and AKI in these regions.

## CONCLUSIONS

Our extensive review and meta-analysis showcased that AKI are common among COVID patients and higher AKI prevalence was observed among ICU patients. In addition, COVID-associated AKI are associated with poor outcome. The diversity of the studies allowed for subgroup analysis for each socioeconomic level and geographic region. We found that the prevalence of COVID-associated AKI differs in across geographic regions. The lack of information on COVID-associated AKI from lower-middle to low-income countries highlight the need for more studies on COVID and AKI in less developed and lower-income countries.

## Additional material

Online Supplementary Document

## References

[1] Palevsky PM COVID-19 and AKI: Where Do We Stand? J Am Soc Nephrol. 2021;32:1029-32. 10.1681/ASN.2020121768 33637516 PMC8259685

[2] Hirsch JS Ng JH Ross DW Sharma P Shah HH Barnett RL Acute kidney injury in patients hospitalized with COVID-19. Kidney Int. 2020;98:209-18. 10.1016/j.kint.2020.05.006 32416116 PMC7229463

[3] Cheng Y Luo R Wang K Zhang M Wang Z Dong L Kidney disease is associated with in-hospital death of patients with COVID-19. Kidney Int. 2020;97:829-38. 10.1016/j.kint.2020.03.005 32247631 PMC7110296

[4] Fu EL Janse RJ de Jong Y van der Endt VHW Milders J van der Willik EM Acute kidney injury and kidney replacement therapy in COVID-19: a systematic review and meta-analysis. Clin Kidney J. 2020;13:550-63. 10.1093/ckj/sfaa160 32897278 PMC7467593

[5] Chan L Chaudhary K Saha A Chauhan K Vaid A Zhao S AKI in Hospitalized Patients with COVID-19. J Am Soc Nephrol. 2021;32:151-60. 10.1681/ASN.2020050615 32883700 PMC7894657

[6] Azeem HA Abdallah H Abdelnaser MM Acute kidney injury in hospitalized patients with COVID-19 (retrospective study). Egypt J Bronchol. 2021;15:3. 10.1186/s43168-021-00056-z

[7] Lumlertgul N Pirondini L Cooney E Kok W Gregson J Camporota L Acute kidney injury prevalence, progression and long-term outcomes in critically ill patients with COVID-19: a cohort study. Ann Intensive Care. 2021;11:123. 10.1186/s13613-021-00914-5 34357478 PMC8343342

[8] Luther T Bülow-Anderberg S Larsson A Rubertsson S Lipcsey M Frithiof R COVID-19 patients in intensive care develop predominantly oliguric acute kidney injury. Acta Anaesthesiol Scand. 2021;65:364-72. 10.1111/aas.13746 33190222 PMC7753792

[9] Hong D Long L Wang AY Lei Y Tang Y Zhao JW Kidney manifestations of mild, moderate and severe coronavirus disease 2019: A retrospective cohort study. Clin Kidney J. 2020;13:340-6. 10.1093/ckj/sfaa083 32695324 PMC7239220

[10] Chang YC Tsai PH Chou YC Lu KC Chang FY Wu CC Biomarkers linked with dynamic changes of renal function in asymptomatic and mildly symptomatic COVID-19 patients. J Pers Med. 2021;11:432. 10.3390/jpm11050432 34069451 PMC8159130

[11] Yuan H Liu J Gao Z Hu F Clinical Features and Outcomes of Acute Kidney Injury in Patients Infected with COVID-19 in Xiangyang, China. Blood Purif. 2021;50:513-9. 10.1159/000513163 33316799 PMC7801975

[12] El Bardai G Houssaini SS Chouhani BA Kabbali N Houssaini TS Is the Use of Dialysis Associated With an Increased Risk of Death in COVID-19-Related Acute Kidney Injury? Cureus. 2022;14:e32373. 10.7759/cureus.32373 36632264 PMC9827414

[13] Kunutsor SK Laukkanen JA Renal complications in COVID-19: a systematic review and meta-analysis. Ann Med. 2020;52:345-53. 10.1080/07853890.2020.1790643 32643418 PMC7877945

[14] Sabaghian T Kharazmi AB Ansari A Omidi F Kazemi SN Hajikhani B COVID-19 and Acute Kidney Injury: A Systematic Review. Frontiers in Medicine. 2022;9:705908. 10.3389/fmed.2022.705908 35445048 PMC9014846

[15] Oliveira CB Lima CAD Vajgel G Campos Coelho AV Sandrin-Garcia P High burden of acute kidney injury in COVID-19 pandemic: systematic review and meta-analysis. J Clin Pathol. 2021;74:796-803. 10.1136/jclinpath-2020-207023 33023941

[16] Moher D Shamseer L Clarke M Ghersi D Liberati A Petticrew M Preferred reporting items for systematic review and meta-analysis protocols (PRISMA-P) 2015 statement. Syst Rev. 2015;4:1. 10.1186/2046-4053-4-1 25554246 PMC4320440

[17] Goyal A, Daneshpajouhnejad P, Hashmi MF, Bashir K. Acute Kidney Injury. Treasure Island, Florida, USA: StatPearls Publishing; 2025. Available: https://www.ncbi.nlm.nih.gov/books/NBK441896/. Accessed: 25 June 2025.

[18] Selby NM Fluck RJ Kolhe NV Taal MW International Criteria for Acute Kidney Injury: Advantages and Remaining Challenges. PLoS Med. 2016;13:e1002122. 10.1371/journal.pmed.1002122 27622526 PMC5021257

[19] Covidence. Covidence – Better systematic review management. 2013. Available: https://www.covidence.org/. Accessed: 29 May 2025.

[20] Wells GASB, O’Connell D, Peterson J, Welch V, Losos M, Tugwell P. The Newcastle-Ottawa Scale (NOS) for assessing the quality of nonrandomised studies in meta-analyses. 2024. Available: https://www.ohri.ca/programs/clinical_epidemiology/oxford.asp. Accessed: 25 June 2025.

[21] Sara B Gerta R Guido S How to perform a meta-analysis with R: a practical tutorial. BMJ Ment Health. 2019;22:153. 31563865 10.1136/ebmental-2019-300117 PMC10231495

[22] Team RCR. A Language and Environment for Statistical Computing. Vienna, Austria: R Foundation for Statistical Computing; 2023.

[23] World Bank. The World by Income and Region 2024. 2024. Available: https://datatopics.worldbank.org/world-development-indicators/the-world-by-income-and-region.html. Accessed: 25 June 2025.

[24] Viechtbauer W Conducting meta-analyses in R with the metafor package. J Stat Softw. 2010;36:1-48. 10.18637/jss.v036.i03

[25] Schwarzer G Chemaitelly H Abu-Raddad LJ Rücker G Seriously misleading results using inverse of Freeman-Tukey double arcsine transformation in meta-analysis of single proportions. Res Synth Methods. 2019;10:476-83. 10.1002/jrsm.1348 30945438 PMC6767151

[26] Röver C Friede T Double arcsine transform not appropriate for meta-analysis. Res Synth Methods. 2022;13:645-8. 10.1002/jrsm.1591 35837800

[27] Barker TH Migliavaca CB Stein C Colpani V Falavigna M Aromataris E Conducting proportional meta-analysis in different types of systematic reviews: a guide for synthesisers of evidence. BMC Med Res Methodol. 2021;21:189. 10.1186/s12874-021-01381-z 34544368 PMC8451728

[28] Sánchez AV Pérez AN Pérez-Carrasco M Sonet MT Buendia YD Ballujera PO Acute kidney injury in critically ill patients with COVID-19: The AKICOV multicenter study in Catalonia. PLoS One. 2023;18:e0284248. 10.1371/journal.pone.0284248 37058544 PMC10104297

[29] Neves PD Sato VAH Mohrbacher S Ferreira BMC Oliveira ÉS Pereira LVB Acute Kidney Injury Due to COVID-19 in Intensive Care Unit: An Analysis From a Latin-American Center. Front Med (Lausanne). 2021;8:620050. 10.3389/fmed.2021.620050 34150790 PMC8211765

[30] Alessandri F Pistolesi V Manganelli C Ruberto F Ceccarelli G Morabito S Acute Kidney Injury and COVID-19: A Picture from an Intensive Care Unit. Blood Purif. 2021;50:767-71. 10.1159/000513153 33412548

[31] Doher MP Torres De Carvalho FR Scherer PF Matsui TN Ammirati AL Caldin Da Silva B Acute Kidney Injury and Renal Replacement Therapy in Critically Ill COVID-19 Patients: Risk Factors and Outcomes: A Single-Center Experience in Brazil. Blood Purif. 2021;50:520-30. 10.1159/000513425 33341806 PMC7801990

[32] Arrestier R Gendreau S Mokrani D Bastard JP Fellahi S Bagate F Acute Kidney Injury in Critically-Ill COVID-19 Patients. J Clin Med. 2022;11:2029. 10.3390/jcm11072029 35407639 PMC8999255

[33] Gündogan K Temel S Ketencioglu BB Rabah B Tutar N Sungur M Acute Kidney Injury in SARS-CoV-2 Infected Critically III Patients. Turk J Nephrol. 2020;29:185-9. 10.5152/turkjnephrol.2020.4448

[34] Ottolina D Zazzeron L Trevisi L Agarossi A Colombo R Fossali T Acute kidney injury (AKI) in patients with Covid-19 infection is associated with ventilatory management with elevated positive end-expiratory pressure (PEEP). J Nephrol. 2022;35:99-111. 10.1007/s40620-021-01100-3 34170508 PMC8226340

[35] Eldaboosy SAM Awad A Farouk A Mahdy W Abdelsalam E Nour SO Acute kidney injury in Coronavirus disease-19 related pneumonia in the intensive care unit: a retrospective multicenter study, Saudi Arabia. Multidiscip Respir Med. 2023;18:895. 10.4081/mrm.2023.895 36936197 PMC10015944

[36] Geri G Darmon M Zafrani L Fartoukh M Voiriot G Le Marec J Acute kidney injury in SARS-CoV2-related pneumonia ICU patients: a retrospective multicenter study. Ann Intensive Care. 2021;11:86. 10.1186/s13613-021-00875-9 34057648 PMC8165682

[37] Schaubroeck H Vandenberghe W Boer W Boonen E Dewulf B Bourgeois C Acute kidney injury in critical COVID-19: a multicenter cohort analysis in seven large hospitals in Belgium. Crit Care. 2022;26:225. 10.1186/s13054-022-04086-x 35879765 PMC9310674

[38] Ounci E Boukabous S Bkiyar H Abda N Bentata Y Housni B Acute kidney injury in critically ill patients with COVID-19: prevalence, risk factors and mortality in eastern Morocco. J Nephrol. 2022;35:2383-6. 10.1007/s40620-022-01401-1 36006607 PMC9406245

[39] Costa RL Sória TC Salles EF Gerecht AV Corvisier MF de Magalhães Menezes MA Acute kidney injury in patients with Covid-19 in a Brazilian ICU: Incidence, predictors and in-hospital mortality. Brazilian Journal of Nephrology. 2021;43:349-58. 10.1590/2175-8239-jbn-2020-0144 33570081 PMC8428632

[40] Joseph A Zafrani L Mabrouki A Azoulay E Darmon M Acute kidney injury in patients with SARS-CoV-2 infection. Ann Intensive Care. 2020;10:117. 10.1186/s13613-020-00734-z 32880774 PMC7471244

[41] Puah SH Cove ME Phua J Kansal A Venkatachalam J Ho VK Association between lung compliance phenotypes and mortality in covid-19 patients with acute respiratory distress syndrome. Ann Acad Med Singap. 2021;50:686-94. 10.47102/annals-acadmedsg.2021129 34625756

[42] Wainstein M Spyrison N Dai DY Ghadimi M Chávez-Iñiguez JS Rizo-Topete L Association of Country Income Level With the Characteristics and Outcomes of Critically Ill Patients Hospitalized With Acute Kidney Injury and COVID-19. Kidney Int Rep. 2023;8:1514-30. 10.1016/j.ekir.2023.05.015 37360820 PMC10219675

[43] Samra T Ramachandran R Kumar V Ray A Mahajan V Ganesan R Biochemical Predictors of Acute Kidney Injury in Critically Ill COVID-19 Patients. OMICS. 2022;26:650-9. 10.1089/omi.2022.0144 36454181

[44] Contrera Rolón NC Varela CF Ferraris A Rojano A Bisso IC Greloni G Characteristics Of Acute Kidney Injury in Adult Patients with Severe Covid-19. Medicina (B Aires). 2022;82:172-80. 35417379

[45] Herzog AL Von Jouanne-Diedrich HK Wanner C Weismann D Schlesinger T Meybohm P COVID-19 and the kidney: A retrospective analysis of 37 critically ill patients using machine learning. PLoS One. 2021;16:e0251932. 10.1371/journal.pone.0251932 34015009 PMC8136725

[46] Silva BC Cordioli RL dos Santos BFC Guerra JCD Rodrigues RD de Souza GM COVID-19-associated coagulopathy and acute kidney injury in critically ill patients. Einstein (Sao Paulo). 2023;21:eAO0119. 10.31744/einstein_journal/2023AO0119 37729353 PMC10501765

[47] Grimaldi D Aissaoui N Blonz G Carbutti G Courcelle R Gaudry S Characteristics and outcomes of acute respiratory distress syndrome related to COVID-19 in Belgian and French intensive care units according to antiviral strategies: the COVADIS multicentre observational study. Ann Intensive Care. 2020;10:131. 10.1186/s13613-020-00751-y 33025225 PMC7537971

[48] Kocayiğit H Özmen Süner K Tomak Y Demır G Kocayığıt İ Yaylaci S Characteristics and outcomes of critically ill patients with covid-19 in Sakarya, Turkey: A single centre cohort study. Turk J Med Sci. 2021;51:440-7. 10.3906/sag-2005-57 33185365 PMC8203134

[49] Hamilton P Hanumapura P Castelino L Henney R Parker K Kumar M Characteristics and outcomes of hospitalised patients with acute kidney injury and COVID-19. PLoS One. 2020;15:e0241544. 10.1371/journal.pone.0241544 33141867 PMC7608889

[50] Thakkar J Chand S Aboodi MS Gone AR Alahiri E Schecter DE Characteristics, Outcomes and 60-Day Hospital Mortality of ICU Patients with COVID-19 and Acute Kidney Injury. Kidney360. 2020;1:1339-44. 10.34067/KID.0004282020 35372894 PMC8815522

[51] Rubin S Orieux A Prevel R Garric A Bats ML Dabernat S Characterization of acute kidney injury in critically ill patients with severe coronavirus disease 2019. Clin Kidney J. 2020;13:354-61. 10.1093/ckj/sfaa099 32695326 PMC7314187

[52] van Lier D Deniau B Santos K Hartmann O Dudoignon E Depret F Circulating dipeptidyl peptidase 3 and bio-adrenomedullin levels are associated with impaired outcomes in critically ill COVID-19 patients: a prospective international multicentre study. ERJ Open Res. 2023;9:00342-2022. 10.1183/23120541.00342-2022 36628268 PMC9571166

[53] Suleyman G Fadel RA Malette KM Hammond C Abdulla H Entz A Clinical Characteristics and Morbidity Associated With Coronavirus Disease 2019 in a Series of Patients in Metropolitan Detroit. JAMA Netw Open. 2020;3:e2012270. 10.1001/jamanetworkopen.2020.12270 32543702 PMC7298606

[54] Qian SZ Hong WD Mao L Lin C Fang Z Pab J Clinical Characteristics and Outcomes of Severe and Critical Patients With 2019 Novel Coronavirus Disease (COVID-19) in Wenzhou: A Retrospective Study. Front Med (Lausanne). 2020;7:552002. 10.3389/fmed.2020.552002 33015108 PMC7500473

[55] Bouguezzi N Ben Saida I Toumi R Meddeb K Ennouri E Bedhiafi A Clinical Features and Outcomes of Acute Kidney Injury in Critically Ill COVID-19 Patients: A Retrospective Observational Study. J Clin Med. 2023;12:5127. 10.3390/jcm12155127 37568528 PMC10419665

[56] Hernández-Cárdenas CM Choreño-Parra JA Torruco-Sotelo C Jurado F Serna-Secundino H Aguilar C Clinical Risk Factors for Mortality Among Critically Ill Mexican Patients With COVID-19. Front Med (Lausanne). 2021;8:699607. 10.3389/fmed.2021.699607 34513872 PMC8429783

[57] Lowe R Ferrari M Nasim-Mohi M Jackson A Beecham R Veighey K Clinical characteristics and outcome of critically ill COVID-19 patients with acute kidney injury: a single centre cohort study. BMC Nephrol. 2021;22:92. 10.1186/s12882-021-02296-z 33722189 PMC7957445

[58] Yakar MN Ergan B Ergün B Küçük M Cantürk A Ergon MC Clinical characteristics and risk factors for 28-day mortality in critically ill patients with covid-19: A retrospective cohort study. Turk J Med Sci. 2021;51:2285-95. 10.3906/sag-2104-356 34461684 PMC8742492

[59] Zheng Y Sun LJ Xu M Pan J Zhang YT Fang XL Clinical characteristics of 34 COVID-19 patients admitted to intensive care unit in Hangzhou, China. J Zhejiang Univ Sci B. 2020;21:378-87. 10.1631/jzus.B2000174 32425003 PMC7238397

[60] Martínez-Rueda AJ Álvarez RD Méndez-Pérez RA Fernández-Camargo DA Gaytan-Arocha JE Berman-Parks N Community- And Hospital-Acquired Acute Kidney Injury in COVID-19: Different Phenotypes and Dismal Prognosis. Blood Purif. 2021;50:931-41. 10.1159/000513948 33744901 PMC8089414

[61] Yildirim M Halacli B Pektezel MY Er B Geldigitti IT Tok G Comparison of critically ill COVID-19 and influenza patients with acute respiratory failure. Acute Crit Care. 2022;37:168-76. 10.4266/acc.2021.00920 35280038 PMC9184989

[62] Bhatraju PK Morrell ED Zelnick L Sathe NA Chai XY Sakr SS Comparison of host endothelial, epithelial and inflammatory response in ICU patients with and without COVID-19: a prospective observational cohort study. Crit Care (Fullerton). 2021;25:148. 10.1186/s13054-021-03547-z 33874973 PMC8054255

[63] Wasfy SF Wasfey EF Elmaraghy AA AbdelFatah EB Tharwat AI Cystatin C and Neutrophil Gelatin-associated Lipocalin (NGAL) Can Predict Acute Kidney Injury and In-Hospital Mortality in COVID-19 Patients. Journal of Cellular and Molecular Anesthesia. 2022;7:32-9.

[64] Ramos-Santos K Cortes-Telles A Uc-Miam ME Avila-Nava A Lugo R Aké RC Cystatin C is a marker for acute kidney injury, but not for mortality among COVID-19 patients in Mexico. Braz J Infect Dis. 2022;26:102365. 10.1016/j.bjid.2022.102365 35576994 PMC9072838

[65] Palomba H Cubos D Bozza F Zampieri FG Romano TG Development of a Risk Score for AKI onset in COVID-19 Patients: COV-AKI Score. BMC Nephrol. 2023;24:46. 10.1186/s12882-023-03095-4 36859175 PMC9977632

[66] Cai Y Tang C Song Y Li Q Guo S Chen Y Duration of Acute Kidney Injury and In-Hospital Mortality in Elder Patients with Severe COVID-19: A Retrospective Cohort Study. BioMed Res Int. 2022;2022:9929038. 10.1155/2022/9929038 35928914 PMC9345708

[67] El Mouhayyar C Dewald J Cabrales J Tighiouart H Moraco AH Jaber BL Factors Associated with Severity of Acute Kidney Injury and Adverse Outcomes in Critically Ill Patients with COVID-19. Nephron. 2022;146:584-92. 10.1159/000524657 35675790 PMC9393776

[68] Anees M Farooq O Raza M Mumtaz A Frequency and Risk Factors for Acute Kidney Injury in patients with COVID-19. Pak J Med Sci. 2022;38:816-21. 10.12669/pjms.38.4.4980 35634634 PMC9121968

[69] Lascarrou JB Gaultier A Soumagne T Serck N Sauneuf B Piagnerelli M Identifying Clinical Phenotypes in Moderate to Severe Acute Respiratory Distress Syndrome Related to COVID-19: The COVADIS Study. Front Med (Lausanne). 2021;8:632933. 10.3389/fmed.2021.632933 33777977 PMC7991403

[70] Husain-Syed F Vadász I Wilhelm J Walmrath HD Seeger W Birk HW Immunoglobulin deficiency as an indicator of disease severity in patients with COVID-19. American Journal of Physiology - Lung Cellular and Molecular Physiology. 2021;320:L590-L599. 10.1152/ajplung.00359.2020 33237794 PMC8057306

[71] Seetaram NK Kusubi P Gayathri BH Arasingeri G Impact Of Acute Kidney Injury In Covid 19 Infected Patients. J Cardiovasc Dis Res. 2022;13:463-8.

[72] Bobot M Tonon D Peres N Guervilly C Lefèvre F Max H Impact of Dexamethasone and Inhaled Nitric Oxide on Severe Acute Kidney Injury in Critically Ill Patients with COVID-19. J Clin Med. 2022;11:6130. 10.3390/jcm11206130 36294451 PMC9604787

[73] Morieri ML Ronco C Avogaro A Farnia F Shestakova M Zaytseva N In hospital risk factors for acute kidney injury and its burden in patients with Sars-Cov-2 infection: a longitudinal multinational study. Sci Rep. 2022;12:3474. 10.1038/s41598-022-07490-z 35236891 PMC8891366

[74] Mayerhöfer T Perschinka F Klein SJ Peer A Lehner GF Bellmann R Incidence, risk factors and outcome of acute kidney injury in critically ill COVID-19 patients in Tyrol, Austria: a prospective multicenter registry study. J Nephrol. 2023;36:2531-40. 10.1007/s40620-023-01760-3 37837501 PMC10703973

[75] Greco M De Rosa S Boehm F Spano S Aceto R Voza A Kinetics of the Cell Cycle Arrest Biomarkers (TIMP2 and IGFBP7) for the Diagnosis of Acute Kidney Injury in Critically Ill COVID-19 Patients. Diagnostics (Basel). 2023;13:317. 10.3390/diagnostics13020317 36673127 PMC9857893

[76] Hittesdorf E Panzer O Wang D Stevens JS Hastie J Jordan DA Mortality and renal outcomes of patients with severe COVID-19 treated in a provisional intensive care unit. J Crit Care. 2021;62:172-5. 10.1016/j.jcrc.2020.12.012 33385774 PMC7834533

[77] Alser O Mokhtari A Naar L Langeveld K Breen KA El Moheb M Multisystem outcomes and predictors of mortality in critically ill patients with COVID-19: Demographics and disease acuity matter more than comorbidities or treatment modalities. J Trauma Acute Care Surg. 2021;90:880-90. 10.1097/TA.0000000000003085 33891572

[78] Yu Y Xu D Fu S Zhang J Yang X Xu L Patients with COVID-19 in 19 ICUs in Wuhan, China: A cross-sectional study. Crit Care. 2020;24:219. 10.1186/s13054-020-02939-x 32410714 PMC7223395

[79] Solanich X Rigo-Bonnin R Gumucio VD Bastard P Rosain J Philippot Q Pre-existing Autoantibodies Neutralizing High Concentrations of Type I Interferons in Almost 10% of COVID-19 Patients Admitted to Intensive Care in Barcelona. J Clin Immunol. 2021;41:1733-44. 10.1007/s10875-021-01136-x 34570326 PMC8475877

[80] Dushianthan A Abdul N Dmochowski J James I Heesom L Westwood J Predictive Role of Haematological Determinants on Outcomes of Critically Ill COVID-19 Patients Admitted to Intensive Care Unit. Cureus. 2021;13:e16764. 10.7759/cureus.16764 34476137 PMC8403496

[81] Fominskiy EV Scandroglio AM Monti G Calabrò MG Landoni G Dell’acqua A Prevalence, Characteristics, Risk Factors, and Outcomes of Invasively Ventilated COVID-19 Patients with Acute Kidney Injury and Renal Replacement Therapy. Blood Purif. 2021;50:102-9. 10.1159/000508657 32659757 PMC7445373

[82] Rostami Z Mastrangelo G Einollahi B Nemati E Shafiee S Ebrahimi M A Prospective Study on Risk Factors for Acute Kidney Injury and All-Cause Mortality in Hospitalized COVID-19 Patients From Tehran (Iran). Front Immunol. 2022;13:874426. 10.3389/fimmu.2022.874426 35928822 PMC9345117

[83] Fisher M Neugarten J Bellin E Yunes M Stahl L Johns TS AKI in Hospitalized Patients with and without COVID-19: A Comparison Study. J Am Soc Nephrol. 2020;31:2145-57. 10.1681/ASN.2020040509 32669322 PMC7461660

[84] Goffe TK Alemu ZA Derese TN Tilahun RB Tilahun YB Acute Kidney Injury Among Admitted COVID-19 Patients in Addis Ababa, Ethiopia. Int J Nephrol Renovasc Dis. 2023;16:83-92. 10.2147/IJNRD.S402946 36987532 PMC10040157

[85] Mohamed MMB Lukitsch I Torres-Ortiz AE Walker JB Varghese V Hernandez-Arroyo CF Acute Kidney Injury Associated with Coronavirus Disease 2019 in Urban New Orleans. Kidney360. 2020;1:614-22. 10.34067/KID.0002652020 35372932 PMC8815549

[86] Sabaghian T Koomleh AA Nassiri AA Kharazmi AB Khalili S Acute Kidney Injury Outcome in COVID-19 Patients. Iran J Kidney Dis. 2022;16:44-51. 10.52547/ijkd.6610 35271499

[87] Zamoner W Santos CADS Magalhães LE Oliveira PGSD Balbi AL Ponce D Acute Kidney Injury in COVID-19: 90 Days of the Pandemic in a Brazilian Public Hospital. Front Med (Lausanne). 2021;8:622577. 10.3389/fmed.2021.622577 33634152 PMC7900413

[88] Shchepalina A Chebotareva N Akulkina L Brovko M Sholomova V Androsova T Acute Kidney Injury in Hospitalized Patients with COVID-19: Risk Factors and Serum Biomarkers. Biomedicines. 2023;11:1246. 10.3390/biomedicines11051246 37238917 PMC10215395

[89] Chebotareva N Berns S Berns A Androsova T Lebedeva M Moiseev S Acute kidney injury and mortality in coronavirus disease 2019: Results from a cohort study of 1,280 patients. Kidney Res Clin Pract. 2021;40:241-9. 10.23876/j.krcp.20.128 34078024 PMC8237114

[90] Kolhe NV Fluck RJ Selby NM Taal MW Acute kidney injury associated with COVID-19: A retrospective cohort study. PLoS Med. 2020;17:e1003406. 10.1371/journal.pmed.1003406 33125416 PMC7598516

[91] Wan YI Bien Z Apea VJ Orkin CM Dhairyawan R Kirwan CJ Acute kidney injury in COVID-19: Multicentre prospective analysis of registry data. Clin Kidney J. 2021;14:2356-64. 10.1093/ckj/sfab071 34751235 PMC8083651

[92] Magalhães LE de Oliveira PGS Favarin AJ Yuasa BK Cardoso PA Zamoner W Acute kidney injury in coronavirus infectious disease: a study of incidence, risk factors, and prognosis during the first wave of the disease in Brazil. Int Urol Nephrol. 2023;55:1501-8. 10.1007/s11255-022-03454-4 36583822 PMC9801153

[93] Kanbay M Medetalibeyoglu A Kanbay A Cevik E Tanriover C Baygul A Acute kidney injury in hospitalized COVID-19 patients. Int Urol Nephrol. 2022;54:1097-104. 10.1007/s11255-021-02972-x 34410587 PMC8374419

[94] Gameiro J Fonseca JA Oliveira J Marques F Bernardo J Costa C Acute kidney injury in hospitalized patients with COVID-19: A Portuguese cohort. Nefrologia. 2021;41:689-698. 10.1016/j.nefro.2021.04.002 36165158 PMC8800378

[95] Abbasi MSR Masud M Sultan K Rehman U Nasim S Manzoor R Acute kidney injury in patients hospitalized with COVID-19 in a Tertiary Care Hospital of Islamabad. Medical Forum Monthly. 2021;32:81-5.

[96] de Almeida DC Franco MDCP Santos DRPD Santos MC Maltoni IS Mascotte F Acute kidney injury: Incidence, risk factors, and outcomes in severe COVID-19 patients. PLoS One. 2021;16:e0251048. 10.1371/journal.pone.0251048 34033655 PMC8148326

[97] Tan LS Huang XY Wang YF Jia Y Pang QL Zhang WX Association of acute kidney injury and clinical outcomes in patients with COVID-19 in Shenzhen, China: a retrospective cohort study. Am J Transl Res. 2020;12:6931-40. 33194083 PMC7653606

[98] Jewell PD Bramham K Galloway J Post F Norton S Teo J COVID-19-related acute kidney injury; incidence, risk factors and outcomes in a large UK cohort. BMC Nephrol. 2021;22:359. 10.1186/s12882-021-02557-x 34719384 PMC8557997

[99] Xu Z Zhang Y Zhang C Xiong F Zhang J Xiong J Clinical Features and Outcomes of COVID-19 Patients with Acute Kidney Injury and Acute Kidney Injury on Chronic Kidney Disease. Aging Dis. 2022;13:884-98. 10.14336/AD.2021.1125 35656097 PMC9116918

[100] Gadhiya KP Hansrivijit P Gangireddy M Goldman JD Clinical characteristics of hospitalised patients with COVID-19 and the impact on mortality: A single-network, retrospective cohort study from Pennsylvania state. BMJ Open. 2021;11:e042549. 10.1136/bmjopen-2020-042549 37579258 PMC8039219

[101] Soh TV Dzawani M Noorlina N Nik F Norazmi A Clinical characteristics of severe acute respiratory syndrome Coronavirus 2 (SARS-CoV2) patients in Hospital Tengku Ampuan Afzan. Med J Malaysia. 2020;75:479-84. 32918413

[102] Hong KS Lee KH Chung JH Shin KC Choi EY Jin HJ Clinical features and outcomes of 98 patients hospitalized with sars-cov-2 infection in daegu, south korea: A brief descriptive study. Yonsei Med J. 2020;61:431-7. 10.3349/ymj.2020.61.5.431 32390367 PMC7214108

[103] Mogga P Venkatraman S Rajagopalan U Rajagopalan P Radhan P Maithrayie K Correlation of AKI with risk factors, ventilatory support, renal replacement therapy in a cohort of COVID-19 patients. Indian J Nephrol. 2022;32:348-58. 10.4103/ijn.ijn_350_21 35967536 PMC9365008

[104] Hardenberg JB Stockmann H Aigner A Gotthardt I Enghard P Hinze C Critical Illness and Systemic Inflammation Are Key Risk Factors of Severe Acute Kidney Injury in Patients With COVID-19. Kidney Int Rep. 2021;6:905-15. 10.1016/j.ekir.2021.01.011 33817450 PMC8007085

[105] Mousavi Movahed SM Akhavizadegan H Dolatkhani F Nejadghaderi SA Aghajani F Faghir Gangi M Different incidences of acute kidney injury (AKI) and outcomes in COVID-19 patients with and without non-azithromycin antibiotics: A retrospective study. J Med Virol. 2021;93:4411-9. 10.1002/jmv.26992 33792956 PMC8251081

[106] Yildirim C Ozger HS Yasar E Tombul N Gulbahar O Yildiz M Early predictors of acute kidney injury in COVID-19 patients. Nephrology (Carlton). 2021;26:513-21. 10.1111/nep.13856 33502771 PMC8014704

[107] Shahbazian H Tafazoli M Nia LS Ghorbani A Halili SA Mehr FJ Evaluation of mortality of COVID-19 patients with acute kidney injury (AKI) in comparison to the non-AKI patients. J Nephropathol. 2023;12:e18376.

[108] Pitre T Dong AHT Jones A Kapralik J Cui S Mah J Incidence and Outcomes of Acute Kidney Injury in Patients Admitted to Hospital With COVID-19: A Retrospective Cohort Study. Can J Kidney Health Dis. 2021;8:20543581211027759. 10.1177/20543581211027759 34290876 PMC8278450

[109] El-Sayed EE Allayeh AK Salem AA Omar SM Zaghlol SM Abd-Elmaguid HM Incidence of acute kidney injury among COVID-19 patients in Egypt. Ren Replace Ther. 2021;7:32. 10.1186/s41100-021-00356-6 34150333 PMC8200786

[110] Alfano G Ferrari A Fontana F Mori G Magistroni R Meschiari M Incidence, risk factors and outcome of acute kidney injury (AKI) in patients with COVID-19. Clin Exp Nephrol. 2021;25:1203-14. 10.1007/s10157-021-02092-x 34196877 PMC8245663

[111] Kilis-Pstrusinska K Akutko K Braksator J Dancewicz A Grosman-dziewiszek P Jamer T Kidney dysfunction and its progression in patients hospitalized duo to covid-19: Contribution to the clinical course and outcomes. J Clin Med. 2021;10:5522. 10.3390/jcm10235522 34884225 PMC8658310

[112] Lu JY Babatsikos I Fisher MC Hou W Duong TQ Longitudinal Clinical Profiles of Hospital vs. Community-Acquired Acute Kidney Injury in COVID-19. Front Med (Lausanne). 2021;8:647023. 10.3389/fmed.2021.647023 34124089 PMC8193058

[113] Allemailem KS Almatroudi A Khan AA Rahmani AH Almarshad IS Alekezem FS Manifestations of renal system involvement in hospitalized patients with COVID-19 in Saudi Arabia. PLoS One. 2021;16:e0253036. 10.1371/journal.pone.0253036 34264954 PMC8282026

[114] Ng JH Hirsch JS Hazzan A Wanchoo R Shah HH Malieckal DA Outcomes Among Patients Hospitalized With COVID-19 and Acute Kidney Injury. Am J Kidney Dis. 2021;77:204-15.e1. 10.1053/j.ajkd.2020.09.002 32961245 PMC7833189

[115] Sjöström A Markgren PO Hansson M Prognostic potential of creatinine and Cystatin C in COVID-19–a retrospective cohort study from Karolinska University Hospital. Scand J Clin Lab Invest. 2023;83:251-7. 10.1080/00365513.2023.2210291 37167478

[116] Barman HA Atici A Sahin I Alici G Aktas Tekin E Baycan ÖF Prognostic significance of cardiac injury in COVID-19 patients with and without coronary artery disease. Coron Artery Dis. 2021;32:359-66. 10.1097/MCA.0000000000000914 32568741 PMC7365584

[117] See YP Young BE Ang LW Ooi XY Chan CP Looi WL Risk Factors for Development of Acute Kidney Injury in COVID-19 Patients: A Retrospective Observational Cohort Study. Nephron. 2021;145:256-64. 10.1159/000514064 33780937 PMC8089436

[118] Contreras-Villamizar K Barbosa O Muñoz AC Suárez JS González CA Vargas DC Risk factors associated with acute kidney injury in a cohort of hospitalized patients with COVID-19. BMC Nephrol. 2023;24:140. 10.1186/s12882-023-03172-8 37217840 PMC10201026

[119] Bjornstad EC Cutter G Guru P Menon S Aldana I House S SARS-CoV-2 infection increases risk of acute kidney injury in a bimodal age distribution. BMC Nephrol. 2022;23:63. 10.1186/s12882-022-02681-2 35144572 PMC8831033

[120] Rahimzadeh H Kazemian S Rahbar M Farrokhpour H Montazeri M Kafan S The Risk Factors and Clinical Outcomes Associated with Acute Kidney Injury in Patients with COVID-19: Data from a Large Cohort in Iran. Kidney Blood Press Res. 2021;46:620-8. 10.1159/000517581 34315161 PMC8450864

[121] Bernardo J Gonçalves J Gameiro J Oliveira J Marques F Duarte I The impact of transient and persistent acute kidney injury in hospital mortality in COVID-19 patients. Brazilian Journal of Nephrology. 2022;44:310-20. 10.1590/2175-8239-jbn-2021-0123 34874052 PMC9518614

[122] Fernández P Saad EJ Barrionuevo AD Marucco FA Heredia MC Barra AT The incidence, risk factors and impact of acute kidney injury in hospitalized patients due to covid-19. Medicina (B Aires). 2021;81:922-30. 34875589

[123] Cheng Y Luo R Wang X Wang K Zhang N Zhang M The incidence, risk factors, and prognosis of acute kidney injury in adult patients with coronavirus disease 2019. Clin J Am Soc Nephrol. 2020;15:1394-402. 10.2215/CJN.04650420 32963018 PMC7536762

[124] Kim SG Han CH Yu SB Lee H Kwon S Kim Y Trajectory of AKI and hospital mortality among patients with COVID-19. Ren Fail. 2023;45:2177086. 10.1080/0886022X.2023.2177086 36876658 PMC10013401

[125] Bravi CA Cazzaniga W Simonini M Larcher A Messaggio E Zagato L Acute Kidney Injury at Hospital Admission for SARS-CoV-2 Infection as a Marker of Poor Prognosis: Clinical Implications for Triage Risk Stratification. Kidney Blood Press Res. 2022;47:147-50. 10.1159/000518271 35158352 PMC8805049

[126] Taher A Alalwan AA Naser N Alsegai O Alaradi A Acute Kidney Injury in COVID-19 Pneumonia: A Single-Center Experience in Bahrain. Cureus. 2020;12:e9693. 10.7759/cureus.9693 32802627 PMC7425827

[127] Yan J Wang J Ding L Liu S Zhan Y Lu J Adaptive immune dysfunction in patients with COVID-19 and impaired kidney function during the omicron surge. Clin Immunol. 2023;248:109271. 10.1016/j.clim.2023.109271 36806705 PMC9938757

[128] Lim JH Cho JH Jeon Y Kim JH Lee GY Jeon S Adverse impact of renin-angiotensin system blockade on the clinical course in hospitalized patients with severe COVID-19: a retrospective cohort study. Sci Rep. 2020;10:20250. 10.1038/s41598-020-76915-4 33219294 PMC7680105

[129] Kutluhan MA Taş A Şahin A Ürkmez A Topaktas R Ataç Ö Assessment of clinical features and renal functions in Coronavirus disease-19: A retrospective analysis of 96 patients. Int J Clin Pract. 2020;74:e13636. 10.1111/ijcp.13636 32894811

[130] Parmar P James A Rosengarten S Oommen A Joseph MA Wilson C COVID-19 clinical course and outcomes in a predominantly black, vulnerable patient population in New York City. Int J Acad Med. 2021;7:81-8. 10.4103/IJAM.IJAM_116_20

[131] Gómez-Belda AB Fernández-Garcés M Mateo-Sanchis E Madrazo M Carmona M Piles-Roger L COVID-19 in older adults: What are the differences with younger patients? Geriatr Gerontol Int. 2021;21:60-5. 10.1111/ggi.14102 33264816 PMC7753273

[132] Pawelka E Karolyi M Mader T Omid S Kelani H Baumgartner S COVID-19 is not “just another flu”: a real-life comparison of severe COVID-19 and influenza in hospitalized patients in Vienna, Austria. Infection. 2021;49:907-16. 10.1007/s15010-021-01610-z 33983624 PMC8117126

[133] Zhou Y Lv L Yao R Cardiac injury on admission linked to worse outcomes in hospitalized COVID-19 patients. Vojnosanit Pregl. 2022;79:539-47. 10.2298/VSP210602012Z

[134] Alshaikh MK Alotair H Alnajjar F Sharaf H Alhafi B Alashgar L Cardiovascular Risk Factors Among Patients Infected with COVID-19 in Saudi Arabia. Vasc Health Risk Manag. 2021;17:161-8. 10.2147/VHRM.S300635 33907410 PMC8071203

[135] Esposito P Russo E Picciotto D Cappadona F Battaglia Y Traverso GB Changes of Acute Kidney Injury Epidemiology during the COVID-19 Pandemic: A Retrospective Cohort Study. J Clin Med. 2022;11:3349. 10.3390/jcm11123349 35743418 PMC9225342

[136] Nabors C Sridhar A Hooda U Lobo SA Levine A Frishman WH Characteristics and Outcomes of Patients 80 Years and Older Hospitalized With Coronavirus Disease 2019 (COVID-19). Cardiol Rev. 2021;29:39-42. 10.1097/CRD.0000000000000368 33136582

[137] Chen Q Wang L Li C Hu W Fan Y Chen Z Chronic cardio-metabolic disease increases the risk of worse outcomes among hospitalized patients with covid-19: A multicenter, retrospective, and real-world study. J Am Heart Assoc. 2021;10:e018451. 10.1161/JAHA.120.018451 34096317 PMC8477891

[138] Tabbakh TA Alhashemi HH Alharbi K Qanash S Alzahrani MS Saati A Clinical Characteristics, Complications, and Predictors of Poor Outcome Among Hospitalized Adult COVID-19 Patients: A Retrospective Cohort Study. Cureus. 2022;14:e28953. 10.7759/cureus.28953 36111328 PMC9462886

[139] Khan Chachar AZ Khan K Khan AA Hasan KMI Zia MA Siddique N Clinical and Demographic Characteristics Including Comorbidities and Their Outcomes Among Patients Hospitalized With COVID-19 in Four Tertiary Care Hospitals Across Lahore. Cureus. 2021;13:e12663. 33604203 10.7759/cureus.12663 PMC7880821

[140] Nasir N Habib K Khanum I Khan N Muhammad ZA Mahmood SF Clinical characteristics and outcomes of COVID-19: Experience at a major tertiary care center in Pakistan. J Infect Dev Ctries. 2021;15:480-9. 10.3855/jidc.14345 33956647

[141] Lee YR Kang MK Song JE Kim HJ Kweon YO Tak WY Clinical outcomes of coronavirus disease 2019 in patients with pre-existing liver diseases: A multicenter study in south korea. Clin Mol Hepatol. 2020;26:562-76. 10.3350/cmh.2020.0126 33053932 PMC7641571

[142] Cataño-Correa JC Cardona-Arias JA Porras-Mancilla JP Tabares-García M Comparison of Survival and Clinical Profile of Adults with COVID-19 Hospitalized in Two Clinics in Medellin, Colombia. Curr Clin Microbiol Rep. 2022;9:11-19. 10.1007/s40588-022-00179-x 35433195 PMC8993667

[143] Li J Guo T Dong D Zhang X Chen X Feng Y Defining heart disease risk for death in COVID-19 infection. QJM. 2020;113:876-82. 10.1093/qjmed/hcaa246 32790836 PMC7454913

[144] Chen L Liu S Tian J Pan H Liu Y Hu J Disease progression patterns and risk factors associated with mortality in deceased patients with COVID-19 in Hubei Province, China. Immun Inflamm Dis. 2020;8:584-94. 10.1002/iid3.343 32857453 PMC7461240

[145] Tzoulis P Waung JA Bagkeris E Hussein Z Biddanda A Cousins J Dysnatremia is a Predictor for Morbidity and Mortality in Hospitalized Patients with COVID-19. J Clin Endocrinol Metab. 2021;106:1637-48. 10.1210/clinem/dgab107 33624101 PMC7928894

[146] Pode Shakked N de Oliveira MHS Cheruiyot I Benoit JL Plebani M Lippi G Early prediction of COVID-19-associated acute kidney injury: Are serum NGAL and serum Cystatin C levels better than serum creatinine? Clin Biochem. 2022;102:1-8. 10.1016/j.clinbiochem.2022.01.006 35093314 PMC8801397

[147] Liu J Shen Y Wen Z Xu Q Wu Z Feng H Efficacy of Thymosin Alpha 1 in the Treatment of COVID-19: A Multicenter Cohort Study. Front Immunol. 2021;12:673693. 10.3389/fimmu.2021.673693 34408744 PMC8366398

[148] Shah C Grando DJ Rainess RA Ayad L Gobran E Benson P Factors associated with increased mortality in hospitalized COVID-19 patients. Ann Med Surg (Lond). 2020;60:308-13. 10.1016/j.amsu.2020.10.071 33169090 PMC7641593

[149] Rashidi H Amiri F Abaforush F Mehraban Z Pouladzadeh M Sedaghat A Frequency of Diabetes Mellitus and Newly Diagnosed Hyperglycemia and Their Impacts on Hospitalized COVID-19 Patients. Shiraz E Med J. 2023;24:161. 10.5812/semj-130154

[150] Spoto S Agrò FE Sambuco F Travaglino F Valeriani E Fogolari M High value of mid-regional proadrenomedullin in COVID-19: A marker of widespread endothelial damage, disease severity, and mortality. J Med Virol. 2021;93:2820-7. 10.1002/jmv.26676 33200824 PMC7753433

[151] Albaba I Chopra A Al-Tarbasheh AH Feustel PJ Mustafa M Oweis J Incidence, Risk Factors, and Outcomes of Rhabdomyolysis in Hospitalized Patients With COVID-19 Infection. Cureus. 2021;13:e19802. 10.7759/cureus.19802 34956789 PMC8693832

[152] Trabulus S Karaca C Balkan II Dincer MT Murt A Ozcan SG Kidney function on admission predicts in-hospital mortality in COVID-19. PLoS One. 2020;15:e0238680. 10.1371/journal.pone.0238680 32881976 PMC7470363

[153] Al Oweidat K Al-Am R Saleh MY Albtoosh AS Toubasi AA Ribie MK Mortality, Intensive Care Unit Admission, and Intubation among Hospitalized Patients with COVID-19: A One-Year Retrospective Study in Jordan. J Clin Med. 2023;12:2651. 10.3390/jcm12072651 37048734 PMC10094820

[154] Ababneh MJ Al-Kasasbeh A Jarrah M Malkawi L Sanduka O Smadi AM Myocardial injury and its correlation to mortality in hospitalized COVID-19 patients: A retrospective cohort study. Front Cardiovasc Med. 2022;9:1039655. 10.3389/fcvm.2022.1039655 36505360 PMC9726781

[155] Imam Z Odish F Gill I O’Connor D Armstrong J Vanood A Older age and comorbidity are independent mortality predictors in a large cohort of 1305 COVID-19 patients in Michigan, United States. J Intern Med. 2020;288:469-76. 10.1111/joim.13119 32498135 PMC7300881

[156] Richardson S Hirsch JS Narasimhan M Crawford JM McGinn T Davidson KW Presenting Characteristics, Comorbidities, and Outcomes among 5700 Patients Hospitalized with COVID-19 in the New York City Area. JAMA. 2020;323:2052-9. 10.1001/jama.2020.6775 32320003 PMC7177629

[157] Erben Y Marquez CP Prudencio M Fortich S Gendron T Sanghavi D Race affects adverse outcomes of deep vein thrombosis, pulmonary embolism, and acute kidney injury in coronavirus disease 2019 hospitalized patients. J Vasc Surg Venous Lymphat Disord. 2023;11:19-24.e3. 10.1016/j.jvsv.2022.05.019 36100130 PMC9463072

[158] Lanzani C Simonini M Arcidiacono T Messaggio E Bucci R Betti P Role of blood pressure dysregulation on kidney and mortality outcomes in COVID-19. Kidney, blood pressure and mortality in SARS-CoV-2 infection. J Nephrol. 2021;34:305-14. 10.1007/s40620-021-00997-0 33656707 PMC7926195

[159] Bugiardini R Nava S Caramori G Yoon J Badimon L Bergami M Sex differences and disparities in cardiovascular outcomes of COVID-19. Cardiovasc Res. 2023;119:1190-201. 10.1093/cvr/cvad011 36651866

[160] Sun S Annadi RR Chaudhri I Munir K Hajagos J Saltz J Short- and Long-Term Recovery after Moderate/Severe AKI in Patients with and without COVID-19. Kidney360. 2021;3:242-57. 10.34067/KID.0005342021 35373118 PMC8967640

[161] Piani F Di Salvo E Landolfo M Saracino IM Agnoletti D Borghi C Statin therapy may protect against acute kidney injury in patients hospitalized for interstitial SARS-CoV2 pneumonia. Nutr Metab Cardiovasc Dis. 2023;33:227-31. 10.1016/j.numecd.2022.10.005 36411214 PMC9576907

[162] Skwiersky S Rosengarten S Meisel T MacAluso F Chang M Thomson A Sugar is not always sweet: exploring the relationship between hyperglycemia and COVID-19 in a predominantly African American population. BMJ Open Diabetes Res Care. 2022;10:e002692. 10.1136/bmjdrc-2021-002692 36002176 PMC9412045

[163] Moledina DG Simonov M Yamamoto Y Alausa J Arora T Biswas A The Association of COVID-19 With Acute Kidney Injury Independent of Severity of Illness: A Multicenter Cohort Study. Am J Kidney Dis. 2021;77:490-9.e1. 10.1053/j.ajkd.2020.12.007 33422598 PMC7791318

[164] Hasegawa T Nakagawa A Suzuki K Yamashita K Yamashita S Iwanaga N Type 1 inflammatory endotype relates to low compliance, lung fibrosis, and severe complications in COVID-19. Cytokine. 2021;148:155618. 10.1016/j.cyto.2021.155618 34127355 PMC8180668

[165] Weiss R Von Groote T Ostermann M Lumlertgul N Weerapolchai K Garcia MIM The Role of Cell Cycle Arrest Biomarkers for Predicting Acute Kidney Injury in Critically Ill COVID-19 Patients: A Multicenter, Observational Study. Crit Care Med. 2023;51:992-1000. 10.1097/CCM.0000000000005853 36975308 PMC10335735

[166] Ouahmi H Courjon J Morand L François J Bruckert V Lombardi R Proteinuria as a Biomarker for COVID-19 Severity. Front Physiol. 2021;12:611772. 10.3389/fphys.2021.611772 33767630 PMC7985082

[167] Gasparini M Khan S Patel JM Parekh D Bangash MN Stϋmpfle R Renal impairment and its impact on clinical outcomes in patients who are critically ill with COVID-19: a multicentre observational study. Anaesthesia. 2021;76:320-6. 10.1111/anae.15293 33948938

[168] Renberg M Jonmarker O Kilhamn N Rimes-Stigare C Bell M Hertzberg D Renal resistive index is associated with acute kidney injury in COVID-19 patients treated in the intensive care unit. Ultrasound J. 2021;13:3. 10.1186/s13089-021-00203-z 33544258 PMC7863038

[169] Molina Barragan AM Pardo E Galichon P Hantala N Gianinazzi AC Darrivere L Sars-cov-2 renal impairment in critical care: An observational study of 42 cases (kidney covid). J Clin Med. 2021;10:1571. 10.3390/jcm10081571 33917886 PMC8068224

[170] Choron RL Butts CA Bargoud C Krumrei N Teichman AL Schroeder M Surgeons in surge - the versatility of the acute care surgeon: outcomes of COVID-19 ICU patients in a community hospital where all ICU patients are managed by surgical intensivists. Trauma Surg Acute Care Open. 2020;5:e000557. 10.1136/tsaco-2020-000557 34192160 PMC7705423

[171] Regolisti G Maggiore U Di Mario F Gentile M Benigno GD Gandolfini I The Association of New-Onset Acute Kidney Injury and Mortality in Critically Ill Patients With COVID-19 With Less Severe Clinical Conditions at Admission: A Moderation Analysis. Front Med (Lausanne). 2022;9:799298. 10.3389/fmed.2022.799298 35372447 PMC8971281

[172] Al Abri SY Burad J Al Wahaibi MM The Incidence of Acute Kidney Injury (AKI) in Critically Ill COVID-19 Patients: A Single-Center Retrospective Cohort Study at a Tertiary Level Hospital in Oman. Cureus. 2023;15:e40340. 10.7759/cureus.40340 37456444 PMC10338890

[173] Maniero C Patel D Pavithran A Naran P Ng FL Prowle J A retrospective cohort study of risk factors and outcomes in older patients admitted to an inner-city geriatric unit in London during first peak of COVID-19 pandemic. Ir J Med Sci. 2022;191:1037-45. 10.1007/s11845-021-02679-z 34228265 PMC8258277

[174] Bhasin B Veitla V Dawson AZ Garacci Z Sturgill D Ozieh MN AKI in Hospitalized Patients with COVID-19 and Seasonal Influenza: A Comparative Analysis. Kidney360. 2021;2:619-28. 10.34067/KID.0007322020 35373047 PMC8791326

[175] Anvar MI Bhaskar BS Chand KN Kalaburgi RA Shaik RA Determinants of Mortality in Patients with Acute Kidney Injury Caused by Coronavirus Disease 2019 Infection in a Tertiary Care Hospital of South India. Saudi J Kidney Dis Transpl. 2022;33:404-12. 10.4103/1319-2442.385963 37843141

[176] Bandelac L Shah KD Purmessur P Ghazanfar H Nasr R Acute Kidney Injury Incidence, Stage, and Recovery in Patients with COVID-19. Int J Nephrol Renovasc Dis. 2022;15:77-83. 10.2147/IJNRD.S352600 35280117 PMC8906872

[177] Zahid U Ramachandran P Spitalewitz S Alasadi L Chakraborti A Azhar M Acute Kidney Injury in COVID-19 Patients: An Inner City Hospital Experience and Policy Implications. Am J Nephrol. 2020;51:786-96. 10.1159/000511160 33011717 PMC7573899

[178] Narayanan A Cunningham P Mehta M Lang T Hammes M Acute Kidney Injury in Coronavirus Disease and Association with Thrombosis. Am J Nephrol. 2023;54:156-164. 10.1159/000530524 37019091

[179] Fabrizi F Alfieri CM Molinari P Tamborini F Tangredi M Sikharulidze A Acute Kidney Injury in Non-Intensive Care Unit (ICU) Hospitalizations for Coronavirus Disease (COVID-19). Pathogens. 2022;11:1272. 10.3390/pathogens11111272 36365023 PMC9693191

[180] Xu H Garcia-Ptacek S Annetorp M Bruchfeld A Cederholm T Johnson P Acute kidney injury and mortality risk in older adults with COVID-19. J Nephrol. 2021;34:295-304. 10.1007/s40620-021-01022-0 33751497 PMC7982881

[181] Sullivan MK Lees JS Drake TM Docherty AB Oates G Hardwick HE Acute kidney injury in patients hospitalized with COVID-19 from the ISARIC WHO CCP-UK Study: A prospective, multicentre cohort study. Nephrol Dial Transplant. 2022;37:271-84. 10.1093/ndt/gfab303 34661677 PMC8788218

[182] Diebold M Schaub S Landmann E Steiger J Dickenmann M Acute kidney injury in patients with COVID-19: a retrospective cohort study from Switzerland. Swiss Med Wkly. 2021;151:w20482. 10.4414/smw.2021.20482 33706383

[183] Scarpioni R Valsania T Albertazzi V Blanco V DeAmicis S Manini A Acute kidney injury, a common and severe complication in hospitalized patients during the COVID-19 pandemic. J Nephrol. 2021;34:1019-24. 10.1007/s40620-021-01087-x 34146335 PMC8214067

[184] Wiley Z Kubes JN Cobb J Jacob JT Franks N Plantinga L Age, Comorbid Conditions, and Racial Disparities in COVID-19 Outcomes. J Racial Ethn Health Disparities. 2022;9:117-23. 10.1007/s40615-020-00934-0 33415702 PMC7790329

[185] Naaraayan A Nimkar A Hasan A Pant S Durdevic M Elenius H Analysis of Male Sex as a Risk Factor in Older Adults With Coronavirus Disease 2019: A Retrospective Cohort Study From the New York City Metropolitan Region. Cureus. 2020;12:e9912. 10.7759/cureus.9912 32974111 PMC7507573

[186] Patel DM Phadke M Dai F Simonov M Dahl NK Kodali R Association of AKI-D with Urinary Findings and Baseline eGFR in Hospitalized COVID-19 Patients. Kidney360. 2021;2:1215-24. 10.34067/KID.0001612021 35369662 PMC8676386

[187] Roberge J Anderson WE Gutnik B Gohs F Palmer P Weaver DJ Association of race and acute kidney injury among patients admitted with Coronavirus disease of 2019 (COVID-19). Clin Nephrol. 2022;97:150-6. 10.5414/CN110568 34642017

[188] Genovesi S Rebora P Occhino G Rossi E Maloberti A Belli M Atrial fibrillation and clinical outcomes in a cohort of hospitalized patients with sars-cov-2 infection and chronic kidney disease. J Clin Med. 2021;10:4108. 10.3390/jcm10184108 34575219 PMC8468274

[189] Filev R Rostaing L Lyubomirova M Bogov B Kalinov K Svinarov D COVID-19 Infection in Chronic Kidney Disease Patients in Bulgaria: Risk Factors for Death and Acute Kidney Injury. J Pers Med. 2022;12:1676. 10.3390/jpm12101676 36294815 PMC9605526

[190] Canto-Costal AC Aguilar-Cantón I Patrón-Iturralde DJ Hussaini N Ramirez L Ziad M COVID-19 in hospitalized patients with and without acute kidney injury. Crit Care Shock. 2023;26:229-36.

[191] Drake TM Riad AM Fairfield CJ Egan C Knight SR Pius R Characterisation of in-hospital complications associated with COVID-19 using the ISARIC WHO Clinical Characterisation Protocol UK: a prospective, multicentre cohort study. Lancet. 2021;398:223-37. 10.1016/S0140-6736(21)00799-6 34274064 PMC8285118

[192] Parker K Hamilton P Hanumapura P Castelino L Murphy M Challiner R Chronic anticoagulation is not associated with a reduced risk of acute kidney injury in hospitalised Covid-19 patients. BMC Nephrol. 2021;22:224. 10.1186/s12882-021-02436-5 34134645 PMC8208381

[193] Gutiérrez-Abejón E Martín-García D Tamayo E Álvarez FJ Herrera-Gómez F Clinical Profile, Pharmacological Treatment, and Predictors of Death Among Hospitalized COVID-19 Patients With Acute Kidney Injury: A Population-Based Registry Analysis. Front Med (Lausanne). 2021;8:657977. 10.3389/fmed.2021.657977 34211984 PMC8240871

[194] Isted A McDonnell AJ Jones E Grundy T Jeyabraba S Ali TO Clinical characteristics and outcomes of 85 intensive care patients with Covid-19 in South London: A single centre observational study. J Intensive Care Soc. 2022;23:34-43. 10.1177/1751143720971541 37593533 PMC10427843

[195] Bell JS James BD Al-Chalabi S Sykes L Kalra PA Green D Community- versus hospital-acquired acute kidney injury in hospitalised COVID-19 patients. BMC Nephrol. 2021;22:269. 10.1186/s12882-021-02471-2 34301204 PMC8299737

[196] Huang ACC Lin SM Chiu TH Chang KW Huang TH Yang TH Comparison of Clinical Characteristics and Outcomes of Hospitalized Patients Infected with the D614G Strain or Alpha Variant of COVID-19 in Taiwan: A Multi-Center Cohort Study. Int J Med Sci. 2022;19:1912-9. 10.7150/ijms.76725 36438919 PMC9682515

[197] Kormann R Jacquot A Alla A Corbel A Koszutski M Voirin P Coronavirus disease 2019: Acute Fanconi syndrome precedes acute kidney injury. Clin Kidney J. 2020;13:362-70. 10.1093/ckj/sfaa109 32695327 PMC7314200

[198] Gómez-Escobar LG Hoffman KL Choi JJ Borczuk A Salvatore S Alvarez-Mulett SL Cytokine signatures of end organ injury in COVID-19. Sci Rep. 2021;11:12606. 10.1038/s41598-021-91859-z 34131192 PMC8206105

[199] Cei F Chiarugi L Brancati S Montini MS Dolenti S Di Stefano D Early reduction of estimated Glomerular Filtration Rate (eGFR) predicts poor outcome in acutely ill hospitalized COVID-19 patients firstly admitted to medical regular wards (eGFR-COV19 study). Biomed Pharmacother. 2022;153:113454. 10.1016/j.biopha.2022.113454 36076568 PMC9300590

[200] Meisel E Efros O Bleier J Halevi TB Segal G Rahav G Folate levels in patients hospitalized with coronavirus disease 2019. Nutrients. 2021;13:812. 10.3390/nu13030812 33801194 PMC8001221

[201] Farooqui MA Almegren A Binrushud SR Alnuwaiser FA Almegren NM Alhamied NA Incidence and Outcome of Acute Kidney Injury Patients Hospitalized With Coronavirus Disease-19 at a Tertiary Care Medical Center in Saudi Arabia. Cureus. 2021;13:e18927. 10.7759/cureus.18927 34812311 PMC8604091

[202] Russo E Esposito P Taramasso L Magnasco L Saio M Briano F Kidney disease and all-cause mortality in patients with COVID-19 hospitalized in Genoa, Northern Italy. J Nephrol. 2021;34:173-83. 10.1007/s40620-020-00875-1 33025516 PMC7538179

[203] Araújo BF Araújo CA Melo M Baptista C Paiva S Paiva I Outcomes of Hospitalized Patients with Type 2 Diabetes and COVID-19: The Impact of Glycaemic Control. Revista Portuguesa de Endocrinologia Diabetes e Metabolismo. 2022;17:33-9. 10.26497/AO210018

[204] Kim SY Hong DY Kim JW Park SO Lee KR Baek KJ Predictive Values of Procalcitonin and Presepsin for Acute Kidney Injury and 30-Day Hospital Mortality in Patients with COVID-19. Medicina (Kaunas). 2022;58:727. 10.3390/medicina58060727 35743990 PMC9229229

[205] Martinot M Eyriey M Gravier S Bonijoly T Kayser D Ion C Predictors of mortality, ICU hospitalization, and extrapulmonary complications in COVID-19 patients. Infect Dis Now. 2021;51:518-25. 10.1016/j.idnow.2021.07.002 34242842 PMC8260549

[206] Karras A Livrozet M Lazareth H Benichou N Hulot JS Fayol A Proteinuria and clinical outcomes in hospitalized covid-19 patients a retrospective single-center study. Clin J Am Soc Nephrol. 2021;16:514-21. 10.2215/CJN.09130620 33661756 PMC8092053

[207] Egbuche O Abe T Nwokike SI Jegede O Mezue K Olanipekun T Racial differences in cardiopulmonary outcomes of hospitalized COVID-19 patients with acute kidney injury. Rev Cardiovasc Med. 2021;22:1667-75. 10.31083/j.rcm2204174 34957809 PMC9054458

[208] Kalligeros M Tashima KT Mylona EK Rybak N Flanigan TP Farmakiotis D Remdesivir use compared with supportive care in hospitalized patients with severe COVID-19: A single-center experience. Open Forum Infect Dis. 2020;7:ofaa319. 10.1093/ofid/ofaa319 33117850 PMC7454852

[209] Li D Ren H Varelmann DJ Sarin P Xu P Wu D Risk assessment for acute kidney injury and death among new COVID-19 positive adult patients without chronic kidney disease: Retrospective cohort study among three US hospitals. BMJ Open. 2022;12:e053635. 10.1136/bmjopen-2021-053635 35190428 PMC8861883

[210] Kim IS Kim DH Lee HW Kim SG Kim YK Kim JK Role of increased neutrophil extracellular trap formation on acute kidney injury in COVID-19 patients. Front Immunol. 2023;14:1122510. 10.3389/fimmu.2023.1122510 37051234 PMC10083414

[211] Paek JH Kim Y Park WY Jin K Hyun M Lee JY Severe acute kidney injury in COVID-19 patients is associated with in-hospital mortality. PLoS One. 2020;15:e0243528. 10.1371/journal.pone.0243528 33296419 PMC7725289

[212] Tejpal A Gianos E Cerise J Hirsch JS Rosen S Kohn N Sex-Based Differences in COVID-19 Outcomes. J Womens Health (Larchmt). 2021;30:492-501. 10.1089/jwh.2020.8974 33885345 PMC8182657

[213] Rola P Doroszko A Trocha M Giniewicz K Kujawa K Skarupski M Sex-Dependent Differences in Predictive Value of the C2HEST Score in Subjects with COVID-19-A Secondary Analysis of the COLOS Study. Viruses. 2022;14:628. 10.3390/v14030628 35337035 PMC8950798

[214] Nakeshbandi M Maini R Daniel P Rosengarten S Parmar P Wilson C The impact of obesity on COVID-19 complications: a retrospective cohort study. Int J Obes (Lond). 2020;44:1832-7. 10.1038/s41366-020-0648-x 32712623 PMC7382318

[215] Randhawa G Syed KA Singh K Kundal SV Oli S Silver M The relationship between obesity, hemoglobin A1c and the severity of COVID-19 at an urban tertiary care center in New York City: A retrospective cohort study. BMJ Open. 2021;11:e044526. 10.1136/bmjopen-2020-044526 33518528 PMC7852070

[216] La Porta E Baiardi P Fassina L Faragli A Perna S Tovagliari F The role of kidney dysfunction in COVID-19 and the influence of age. Sci Rep. 2022;12:8650. 10.1038/s41598-022-12652-0 35606394 PMC9125966

[217] Schnabel K Garam N Ledó N Hajdú N Kóczy Á Takács I Urinary albumin-to-creatinine ratio and serum albumin are predictors of acute kidney injury in non-ventilated COVID-19 patients: a single-center prospective cohort study. Int Urol Nephrol. 2023;55:711-20. 10.1007/s11255-022-03348-5 36127479 PMC9488874

[218] McAdams MC Li M Xu P Gregg LP Patel J Willett DL Using dipstick urinalysis to predict development of acute kidney injury in patients with COVID-19. BMC Nephrol. 2022;23:50. 10.1186/s12882-022-02677-y 35105331 PMC8805668

[219] Avotins L Kroica J Petersons A Zentina D Kravale Z Saulite A eGFRcystatinC/eGFRcreatinine ratio < 0.6 in patients with SARS-CoV-2 pneumonia: a prospective cohort study. BMC Nephrol. 2023;24:269. 10.1186/s12882-023-03315-x 37704948 PMC10500727

[220] Hectors SJ Riyahi S Dev H Krishnan K Margolis DJA Prince MR Multivariate analysis of CT imaging, laboratory, and demographical features for prediction of acute kidney injury in COVID-19 patients: a Bi-centric analysis. Abdom Radiol (NY). 2021;46:1651-8. 10.1007/s00261-020-02823-w 33098478 PMC7584857

[221] Sang L Chen S Zheng X Guan W Zhang Z Liang W The incidence, risk factors and prognosis of acute kidney injury in severe and critically ill patients with COVID-19 in mainland China: a retrospective study. BMC Pulm Med. 2020;20:290. 10.1186/s12890-020-01305-5 33167955 PMC7649893

[222] Liu J Wang T Cai Q Huang D Sun L He Q Acute Kidney Injury and Early Predictive Factors in COVID-19 Patients. Front Med (Lausanne). 2021;8:604242. 10.3389/fmed.2021.604242 34322497 PMC8311118

[223] Dai Y Liu Z Du X Wei H Wu Y Li H Acute Kidney Injury in Hospitalized Patients Infected with COVID-19 from Wuhan, China: A Retrospective Study. BioMed Res Int. 2021;2021:6655185. 10.1155/2021/6655185 33506027 PMC7801940

[224] Li WX Xu W Huang CL Fei L Xie XD Li Q Acute cardiac injury and acute kidney injury associated with severity and mortality in patients with COVID-19. Eur Rev Med Pharmacol Sci. 2021;25:2114-22. 33660831 10.26355/eurrev_202102_25117

[225] Chen X Chen Y Wu C Wei M Xu J Chao YC Coagulopathy is a major extrapulmonary risk factor for mortality in hospitalized patients with COVID-19 with type 2 diabetes. BMJ Open Diabetes Res Care. 2020;8:e001851. 10.1136/bmjdrc-2020-001851 33214191 PMC7677866

[226] Li XQ Liu H Meng Y Yin HY Gao WY Yang X Critical roles of cytokine storm and secondary bacterial infection in acute kidney injury development in COVID-19: A multi-center retrospective cohort study. J Med Virol. 2021;93:6641-52. 10.1002/jmv.27234 34314040 PMC8426723

[227] Peng S Wang HY Sun X Li P Ye Z Li Q Early versus late acute kidney injury among patients with COVID-19 — A multicenter study from Wuhan, China. Nephrol Dial Transplant. 2020;35:2095-102. 10.1093/ndt/gfaa288 33275762 PMC7798799

[228] Chen Z Gao C Yu H Lu L Liu J Chen W Hypophosphatemia is an independent risk factor for AKI among hospitalized patients with COVID-19 infection. Ren Fail. 2021;43:1329-37. 10.1080/0886022X.2021.1979039 34541999 PMC8462927

[229] He L Zhang Q Li Z Shen L Zhang J Wang P Incorporation of Urinary Neutrophil Gelatinase-Associated Lipocalin and Computed Tomography Quantification to Predict Acute Kidney Injury and In-Hospital Death in COVID-19 Patients. Kidney Dis (Basel). 2021;7:120-30. 10.1159/000511403 33824868 PMC7573910

[230] Liu YM Xie J Chen MM Zhang X Cheng X Li H Kidney Function Indicators Predict Adverse Outcomes of COVID-19. Med. 2021;2:38-48.e2. 10.1016/j.medj.2020.09.001 33043313 PMC7531337

[231] Zheng X Yang H Li X Li H Xu L Yu Q Prevalence of kidney injury and associations with critical illness and death in patients with COVID-19. Clin J Am Soc Nephrol. 2020;15:1549-56. 10.2215/CJN.04780420 32943396 PMC7646240

[232] Shang Y Liu T Wei Y Li J Shao L Liu M Scoring systems for predicting mortality for severe patients with COVID-19. EClinicalMedicine. 2020;24:100426. 10.1016/j.eclinm.2020.100426 32766541 PMC7332889

[233] Song XC Zhou XH Cheng JH Zhang WH Shen X Xu H The roles of inactivated vaccines in older patients with infection of Delta variant in Nanjing, China. Aging (Albany NY). 2022;14:4211-9. 10.18632/aging.204085 35585022 PMC9186756

[234] Nlandu Y Mafuta D Sakaji J Brecknell M Engole Y Abatha J Predictors of mortality in COVID-19 patients at Kinshasa Medical Center and a survival analysis: a retrospective cohort study. BMC Infect Dis. 2021;21:1272. 10.1186/s12879-021-06984-x 34930174 PMC8686084

[235] Bashir AM Mukhtar MS Cetinkaya O Fiidow OA Mohamed YG Prevalence of Acute Kidney Injury in Covid-19 Patients-Retrospective Single-Center Study. Infect Drug Resist. 2022;15:1555-60. 10.2147/IDR.S357997 35411159 PMC8994562

[236] Ibrahim OR Oloyede T Gbadamosi H Musa Y Aliu R Bello SO Acute kidney injury in COVID-19: A single-center experience in Nigeria. Anaesth Pain Intensive Care. 2021;25:470-7. 10.35975/apic.v25i4.1567

[237] Sitina M Sramek V Helan M Suk P Prognostic significance of early acute kidney injury in COVID-19 patients requiring mechanical ventilation: a single-center retrospective analysis. Ren Fail. 2023;45:2205954. 10.1080/0886022X.2023.2205954 37133859 PMC10158536

[238] Cruz EG Broca Garcia BE Sandoval DM Gopar-Nieto R Gonzalez Ruiz FJ Gallardo LD Renal Resistive Index as a Predictor of Acute Kidney Injury and Mortality in COVID-19 Critically Ill Patients. Blood Purif. 2022;51:309-16. 10.1159/000517469 34280921 PMC8339011

[239] Ferlicolak L Alkan Tekes I Altintas ND Risk Factors and Effects on Mortality in Critically ill COVID-19 Patients: A Retrospective Cohort Study. J Crit Intensive Care. 2022;13:110-4.

[240] Bülow Anderberg S Lipcsey M Hultström M Eriksson AK Venge P Frithiof R Systemic human neutrophil lipocalin associates with severe acute kidney injury in SARS-CoV-2 pneumonia. J Clin Med. 2021;10:4144. 10.3390/jcm10184144 34575252 PMC8464787

[241] Rocans RP Ozolina A Battaglini D Bine E Birnbaums JV Tsarevskaya A The Impact of Different Ventilatory Strategies on Clinical Outcomes in Patients with COVID-19 Pneumonia. J Clin Med. 2022;11:2710. 10.3390/jcm11102710 35628835 PMC9143826

[242] Anumas S Chueachinda S Tantiyavarong P Pattharanitima P The Prediction Score of Acute Kidney Injury in Patients with Severe COVID-19 Infection. J Clin Med. 2023;12:4412. 10.3390/jcm12134412 37445447 PMC10342471

[243] Küçük M Ergün B Yakar MN Çakici ÖU Yaka E Cömert B The effect of frailty on the development of acute kidney injury in critically-ill geriatric patients with COVID-19. Turk J Med Sci. 2022;52:1495-503. 10.55730/1300-0144.5488 36422488 PMC10395705

[244] Bayrakci N Özkan G Şakaci M Sedef S Erdem İ Tuna N The incidence of acute kidney injury and its association with mortality in patients diagnosed with COVID-19 followed up in intensive care unit. Ther Apher Dial. 2022;26:889-96. 10.1111/1744-9987.13790 34990070

[245] Khan FN Khan Q Bhatti JM Hashmat S Fatima S Mahmood SN AKI and Its Relation with Outcome in Patients with COVID-19. Pak J Med Health Sci. 2022;16:483-6. 10.53350/pjmhs20221612483

[246] Ahmed R Maula KF Ali Z Ismail M Rehman IU Maula SF Acute Kidney Injury and Mortality among Patients with Coronavirus Disease-2019 in Pakistan. Saudi J Kidney Dis Transpl. 2021;32:1764-74. 10.4103/1319-2442.352439 35946291

[247] Medeiros T Guimarães GMC Carvalho FR Alves LS Faustino R Campi-Azevedo AC Acute kidney injury associated to COVID-19 leads to a strong unbalance of circulant immune mediators. Cytokine. 2022;157:155974. 10.1016/j.cyto.2022.155974 35907365 PMC9309102

[248] Casas-Aparicio GA León-Rodríguez I La Barrera CAD González-Navarro M Peralta-Prado AB Luna-Villalobos Y Acute kidney injury in patients with severe COVID-19 in Mexico. PLoS One. 2021;16:e0246595. 10.1371/journal.pone.0246595 33556150 PMC7870064

[249] Romaní L León-Figueroa DA Rafael-Navarro D Barboza JJ Rodriguez-Morales AJ Association between the Use of Antibiotics and the Development of Acute Renal Injury in Patients Hospitalized for COVID-19 in a Hospital in the Peruvian Amazon. J Clin Med. 2022;11:4493. 10.3390/jcm11154493 35956109 PMC9369744

[250] Roozbeh J Hamidianjahromi A Doostkam A Malekmakan L Dorraninejad A COVID-19 Associated Acute Kidney Injury: The Incidence and Associated Factors in Different KDIGO Stages Among the Hospitalized Patients. Iran J Kidney Dis. 2023;17:255-62. 37838935

[251] Bennouar S Bachir Cherif A Kessira A Bennouar DE Abdi S Combined effect of Controlling Nutritional Status and Acute Kidney Injury on severe COVID-19 short-term outcomes. Nutr Clin Metab. 2021;35:144-51. 10.1016/j.nupar.2021.03.001

[252] Algahtani FD Elabbasy MT Alshammari F Atta A El-Fateh AM Ghoniem ME Evolving Risk of Acute Kidney Injury in COVID-19 Hospitalized Patients: A Single Center Retrospective Study. Medicina (Kaunas). 2022;58:443. 10.3390/medicina58030443 35334619 PMC8955925

[253] Sharma H Behera MR Bhadauria DS Khushwaha RS Yachha M Patel MR High mortality and residual kidney damage with Coronavirus disease-19-associated acute kidney injury in northern India. Clin Exp Nephrol. 2022;26:1067-77. 10.1007/s10157-022-02247-4 35804207 PMC9267704

[254] Chávez-Íñiguez JS Cano-Cervantes JH Maggiani-Aguilera P Lavelle-Góngora N Marcial-Meza J Camacho-Murillo EP Mortality and evolution between community and hospital-acquired COVID-AKI. PLoS One. 2021;16:e0257619. 10.1371/journal.pone.0257619 34735451 PMC8568145

[255] Al-Omer DK Outcomes of COVID 19 patients with acute kidney injury in Thi Qar province, south of Iraq. Journal of Population Therapeutics and Clinical Pharmacology. 2023;30:e63-e71.

[256] Ahsan MN Asghar MS Iqbal S Alvi H Akram M Fayyaz B Outcomes of COVID-19 patients with acute kidney injury and longitudinal analysis of laboratory markers during the hospital stay: A multi-center retrospective cohort experience from Pakistan. Medicine (Baltimore). 2023;102:e32919. 10.1097/MD.0000000000032919 36820547 PMC9907899

[257] Thompson JV Meghani NJ Powell BM Newell I Craven R Skilton G Patient characteristics and predictors of mortality in 470 adults admitted to a district general hospital in England with Covid-19. Epidemiol Infect. 2020;148:e285. 10.1017/S0950268820002873 33228824 PMC7729176

[258] Mohammad Y Gireesh S Mahesh E Rajashekar R Gurudev K Karteek U Renal Manifestation of COVID-19 and its Association with Severity of Disease in a Tertiary Care Hospital of South India. Turk J Nephrol. 2022;31:218-24. 10.5152/turkjnephrol.2022.22263

[259] Sundaram S Soni M Annigeri R Urine abnormalities predict acute kidney injury in COVID-19 patients: An analysis of 110 cases in Chennai, South India. Diabetes Metab Syndr. 2021;15:187-91. 10.1016/j.dsx.2020.12.021 33383438 PMC7832278

[260] Xu Z Tang Y Huang Q Fu S Li X Lin B Systematic review and subgroup analysis of the incidence of acute kidney injury (AKI) in patients with COVID-19. BMC Nephrol. 2021;22:52. 10.1186/s12882-021-02244-x 33546616 PMC7863041

[261] Diagnosis and Treatment Protocol for Novel Coronavirus Pneumonia. (Trial Version 7). Chin Med J (Engl). 2020;133:1087-95. 10.1097/CM9.0000000000000819 32358325 PMC7213636

[262] Chen S Yang J Yang W Wang C Bärnighausen T COVID-19 control in China during mass population movements at New Year. Lancet. 2020;395:764-6. 10.1016/S0140-6736(20)30421-9 32105609 PMC7159085

[263] Sun S Xie Z Yu K Jiang B Zheng S Pan X COVID-19 and healthcare system in China: challenges and progression for a sustainable future. Global Health. 2021;17:14. 10.1186/s12992-021-00665-9 33478558 PMC7819629

[264] Chen S Zhang Z Yang J Wang J Zhai X Bärnighausen T Fangcang shelter hospitals: a novel concept for responding to public health emergencies. Lancet. 2020;395:1305-14. 10.1016/S0140-6736(20)30744-3 32247320 PMC7270591

[265] Lyu S Qian C McIntyre A Lee CH One Pandemic, Two Solutions: Comparing the U.S.-China Response and Health Priorities to COVID-19 from the Perspective of “Two Types of Control”. Healthcare (Basel). 2023;11:1848. 10.3390/healthcare11131848 37444682 PMC10341116

[266] Gao J Zhang P China’s Public Health Policies in Response to COVID-19: From an “Authoritarian” Perspective. Front Public Health. 2021;9:756677. 10.3389/fpubh.2021.756677 34976920 PMC8714736

[267] US Centers for Disease Control and Prevention. Infection Control Guidance: SARS-CoV-2. 24 June 2024. Available: https://www.cdc.gov/covid/hcp/infection-control/index.html. Accessed: 30 May 2025.

[268] Lin L Wang X Ren J Sun Y Yu R Li K Risk factors and prognosis for COVID-19-induced acute kidney injury: a meta-analysis. BMJ Open. 2020;10:e042573. 10.1136/bmjopen-2020-042573 33172950 PMC7656886

[269] Susantitaphong P Cruz DN Cerda J Abulfaraj M Alqahtani F Koulouridis I World incidence of AKI: a meta-analysis. Clin J Am Soc Nephrol. 2013;8:1482-93. 10.2215/CJN.00710113 23744003 PMC3805065

[270] Zhang J Pang Q Zhou T Meng J Dong X Wang Z Risk factors for acute kidney injury in COVID-19 patients: an updated systematic review and meta-analysis. Ren Fail. 2023;45:2170809. 10.1080/0886022X.2023.2170809 37021610 PMC10081062

[271] Imrey PB Limitations of Meta-analyses of Studies With High Heterogeneity. JAMA Netw Open. 2020;3:e1919325. 10.1001/jamanetworkopen.2019.19325 31922554

[272] Sabitova A McGranahan R Altamore F Jovanovic N Windle E Priebe S Indicators Associated With Job Morale Among Physicians and Dentists in Low-Income and Middle-Income Countries: A Systematic Review and Meta-analysis. JAMA Network Open. 2020;3:e1913202. 10.1001/jamanetworkopen.2019.13202 31922555 PMC6991249

[273] Luo X Jiang L Du B Wen Y Wang M Xi X A comparison of different diagnostic criteria of acute kidney injury in critically ill patients. Crit Care. 2014;18:R144. 10.1186/cc13977 25005361 PMC4227114

