# Supplementary material for: Global geographic and socioeconomic disparities in COVID-associated acute kidney injury: a systematic review and meta-analysis
Source: J Glob Health. 2025 Jul 25;15:04166. doi: 10.7189/jogh.15.04166 (PMC12290987; doi:10.7189/jogh.15.04166)
Supplement: Online Supplementary Document [file jogh-15-04166-s001.pdf]

Supplement to: Dai D, Gois PF, Simpson D, Hedfi S, Shrapnel S, Pole JD. Global geographic and socioeconomic disparities in COVID-associated acute kidney injury: a systematic review and meta-analysis. J Glob Health. 2025;15:04166.

Supplementary materials

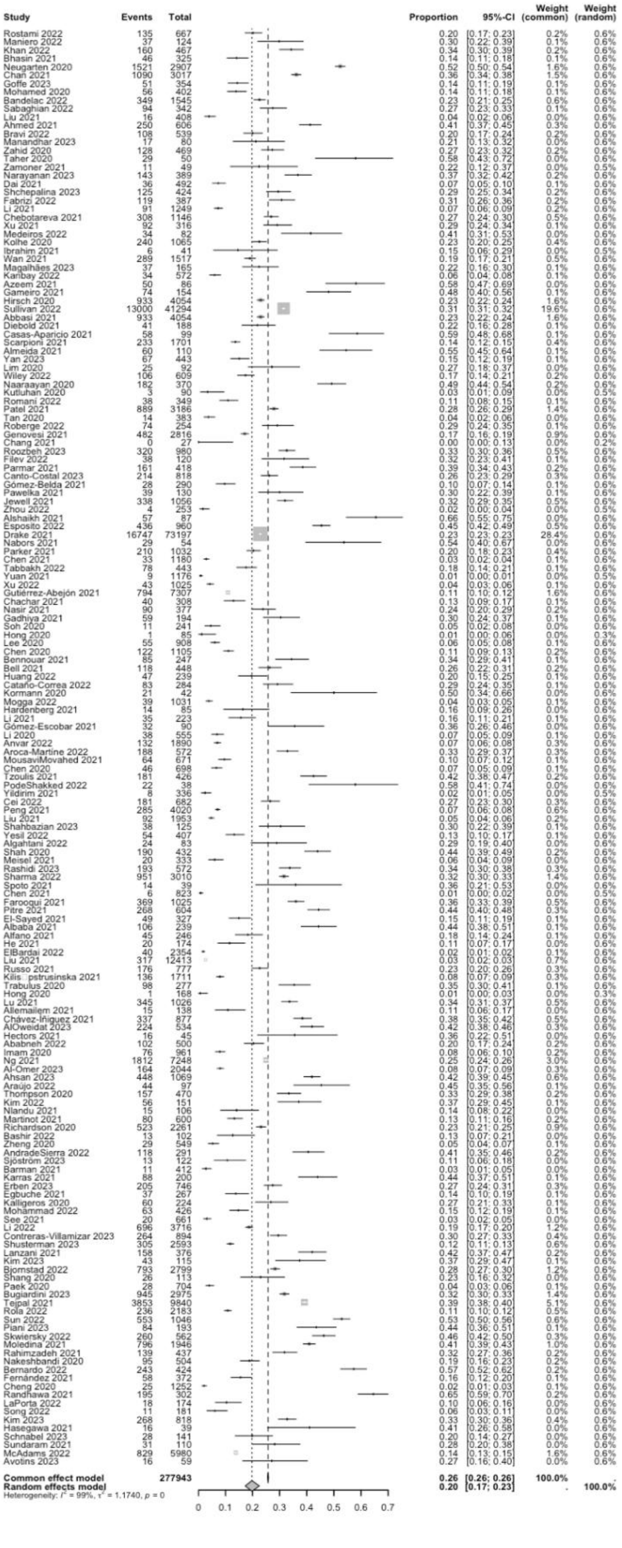

Figure S1. Forest Plot for Non-ICU patients.

Study	Events	Total	Proportion	95%-CI	Weight (common)	Weight (random)
DeLaVegaSánchez 2023	618	1642	0.38	[0.35; 0.40]	2.7%	0.9%
Neves 2021	54	95	0.57	[0.46; 0.67]	0.2%	0.8%
Alessandri 2021	35	61	0.57	[0.44; 0.70]	0.1%	0.8%
Doher 2021	101	201	0.50	[0.43; 0.57]	0.3%	0.9%
Arrestier 2022	45	70	0.64	[0.52; 0.75]	0.1%	0.8%
Gündogan 2020	12	16	0.75	[0.48; 0.93]	0.0%	0.7%
Ottolina 2022	36	101	0.36	[0.26; 0.46]	0.2%	0.8%
Eldaboosy 2023	205	340	0.60	[0.55; 0.66]	0.6%	0.9%
Geri 2021	195	379	0.51	[0.46; 0.57]	0.7%	0.9%
Schaubroeck 2022	1095	1286	0.85	[0.83; 0.87]	1.1%	0.9%
Ounci 2022	210	600	0.35	[0.31; 0.39]	0.9%	0.9%
daCosta 2021	57	102	0.56	[0.46; 0.66]	0.2%	0.9%
Joseph 2020	81	100	0.81	[0.72; 0.88]	0.1%	0.8%
Lumlertgul 2021	240	313	0.77	[0.72; 0.81]	0.4%	0.9%
Puah 2021	55	102	0.54	[0.44; 0.64]	0.2%	0.9%
ISARICCharacterizationGrp 2023	11138	31120	0.36	[0.35; 0.36]	49.4%	0.9%
Samra 2022	69	252	0.27	[0.22; 0.33]	0.3%	0.9%
Rolón 2022	70	162	0.43	[0.35; 0.51]	0.3%	0.9%
Herzog 2021	28	37	0.76	[0.59; 0.88]	0.0%	0.8%
Luther 2021	51	57	0.89	[0.78; 0.96]	0.0%	0.7%
daSilva 2023	13	30	0.43	[0.25; 0.63]	0.1%	0.8%
Grimaldi 2020	231	414	0.56	[0.51; 0.61]	0.7%	0.9%
Kocayığıt 2021	31	103	0.30	[0.21; 0.40]	0.1%	0.8%
Hamilton 2020	210	1032	0.20	[0.18; 0.23]	1.2%	0.9%
Thakkar 2020	224	300	0.75	[0.69; 0.79]	0.4%	0.9%
Rubin 2020	57	71	0.80	[0.69; 0.89]	0.1%	0.8%
vanLier 2023	34	80	0.42	[0.32; 0.54]	0.1%	0.8%
Suleyman 2020	159	355	0.45	[0.40; 0.50]	0.6%	0.9%
Qian 2020	8	37	0.22	[0.10; 0.38]	0.0%	0.8%
Bouguezzi 2023	229	465	0.49	[0.45; 0.54]	0.8%	0.9%
Hernández-Cárdenas 2021	44	67	0.66	[0.53; 0.77]	0.1%	0.8%
Lowe 2021	36	81	0.44	[0.33; 0.56]	0.1%	0.8%
Isted 2022	10	85	0.12	[0.06; 0.21]	0.1%	0.8%
Yakar 2021	139	249	0.56	[0.49; 0.62]	0.4%	0.9%
Zheng 2020	7	34	0.21	[0.09; 0.38]	0.0%	0.7%
Martínez-Rueda 2021	349	1170	0.30	[0.27; 0.33]	1.7%	0.9%
Yildirim 2022	16	54	0.30	[0.18; 0.44]	0.1%	0.8%
Bhatraju 2021	23	78	0.29	[0.20; 0.41]	0.1%	0.8%
Wasfy 2022	25	89	0.28	[0.19; 0.39]	0.1%	0.8%
Ramos-Santos 2022	27	38	0.71	[0.54; 0.85]	0.1%	0.8%
Palomba 2023	376	1131	0.33	[0.31; 0.36]	1.7%	0.9%
Cai 2022	48	107	0.45	[0.35; 0.55]	0.2%	0.9%
ElMouhayyar 2022	371	459	0.81	[0.77; 0.84]	0.5%	0.9%
Anees 2022	90	176	0.51	[0.44; 0.59]	0.3%	0.9%
Lascarrou 2021	225	416	0.54	[0.49; 0.59]	0.7%	0.9%
Husain-Syed 2021	23	62	0.37	[0.25; 0.50]	0.1%	0.8%
Seetaram 2022	166	542	0.31	[0.27; 0.35]	0.8%	0.9%
Bobot 2022	97	164	0.59	[0.51; 0.67]	0.3%	0.9%
Morieri 2022	140	939	0.15	[0.13; 0.17]	0.8%	0.9%
Mayerhöfer 2023	267	1042	0.26	[0.23; 0.28]	1.4%	0.9%
Greco 2023	39	41	0.95	[0.83; 0.99]	0.0%	0.6%
Hittesdorf 2021	76	116	0.66	[0.56; 0.74]	0.2%	0.9%
Alser 2021	164	235	0.70	[0.63; 0.76]	0.3%	0.9%
Yu 2020	57	226	0.25	[0.20; 0.31]	0.3%	0.9%
Solanich 2021	117	275	0.43	[0.37; 0.49]	0.5%	0.9%
Dushianthan 2021	46	71	0.65	[0.53; 0.76]	0.1%	0.8%
Fominskiy 2021	72	96	0.75	[0.65; 0.83]	0.1%	0.8%
Sitina 2023	75	372	0.20	[0.16; 0.25]	0.4%	0.9%
Ouahmi 2021	12	45	0.27	[0.15; 0.42]	0.1%	0.8%
Cruz 2022	22	65	0.34	[0.23; 0.47]	0.1%	0.8%
Gasparini 2021	168	372	0.45	[0.40; 0.50]	0.6%	0.9%
Renberg 2021	23	51	0.45	[0.31; 0.60]	0.1%	0.8%
Ferlicolak 2022	120	206	0.58	[0.51; 0.65]	0.3%	0.9%
Barragan 2021	24	42	0.57	[0.41; 0.72]	0.1%	0.8%
Choron 2020	76	120	0.63	[0.54; 0.72]	0.2%	0.9%
Anderberg 2021	67	103	0.65	[0.55; 0.74]	0.2%	0.8%
Regolisti 2022	42	115	0.37	[0.28; 0.46]	0.2%	0.9%
Rocans 2022	94	530	0.18	[0.15; 0.21]	0.5%	0.9%
AlAbri 2023	158	286	0.55	[0.49; 0.61]	0.5%	0.9%
Anumas 2023	102	215	0.47	[0.41; 0.54]	0.4%	0.9%
Weiss 2023	86	300	0.29	[0.24; 0.34]	0.4%	0.9%
Küçük 2022	84	132	0.64	[0.55; 0.72]	0.2%	0.9%
Bayrakci 2022	88	328	0.27	[0.22; 0.32]	0.4%	0.9%
Sang 2020	92	210	0.44	[0.37; 0.51]	0.4%	0.9%
Rostami 2022	150	330	0.45	[0.40; 0.51]	0.6%	0.9%
Neugarten 2020	382	438	0.87	[0.84; 0.90]	0.3%	0.9%
Chan 2021	745	976	0.76	[0.74; 0.79]	1.2%	0.9%
Goffe 2023	25	48	0.52	[0.37; 0.67]	0.1%	0.8%
Mohamed 2020	105	173	0.61	[0.53; 0.68]	0.3%	0.9%
Sabaghian 2022	10	25	0.40	[0.21; 0.61]	0.0%	0.8%
Zamoner 2021	40	52	0.77	[0.63; 0.87]	0.1%	0.8%
Shchepalina 2023	65	76	0.86	[0.76; 0.93]	0.1%	0.8%
Chebotareva 2021	63	134	0.47	[0.38; 0.56]	0.2%	0.9%
Kolhe 2020	64	96	0.67	[0.56; 0.76]	0.1%	0.8%
Wan 2021	166	338	0.49	[0.44; 0.55]	0.6%	0.9%
Magalhães 2023	124	182	0.68	[0.61; 0.75]	0.3%	0.9%
Kanbay 2022	58	198	0.29	[0.23; 0.36]	0.3%	0.9%
Gameiro 2021	32	38	0.84	[0.69; 0.94]	0.0%	0.7%
Hirsch 2020	1060	1395	0.76	[0.74; 0.78]	1.8%	0.9%
Abbasi 2021	1060	1395	0.76	[0.74; 0.78]	1.8%	0.9%
Almeida 2021	138	168	0.82	[0.75; 0.88]	0.2%	0.9%
Tan 2020	26	34	0.76	[0.59; 0.89]	0.0%	0.8%
Jewell 2021	149	192	0.78	[0.71; 0.83]	0.2%	0.9%
Xu 2022	17	52	0.33	[0.20; 0.47]	0.1%	0.8%
Gadhiya 2021	56	89	0.63	[0.52; 0.73]	0.1%	0.8%
Soh 2020	5	6	0.83	[0.36; 1.00]	0.0%	0.4%
Hong 2020	8	13	0.62	[0.32; 0.86]	0.0%	0.7%
Mogga 2022	47	229	0.21	[0.15; 0.26]	0.3%	0.9%
Hardenberg 2021	103	138	0.75	[0.67; 0.82]	0.2%	0.9%
MousaviMovahed 2021	54	183	0.30	[0.23; 0.37]	0.3%	0.9%
Yildirim 2021	9	12	0.75	[0.43; 0.95]	0.0%	0.6%
Shahbazian 2023	41	69	0.59	[0.47; 0.71]	0.1%	0.8%
Pitre 2021	171	211	0.81	[0.75; 0.86]	0.2%	0.9%
El-Sayed 2021	29	204	0.14	[0.10; 0.20]	0.2%	0.9%
Alfano 2021	24	61	0.39	[0.27; 0.53]	0.1%	0.8%
ElBardai 2022	21	206	0.10	[0.06; 0.15]	0.1%	0.8%
Kilis□pstrusinska 2021	101	247	0.41	[0.35; 0.47]	0.4%	0.9%
Lu 2021	258	298	0.87	[0.82; 0.90]	0.2%	0.9%
Allemailem 2021	33	55	0.60	[0.46; 0.73]	0.1%	0.8%
Ng 2021	2042	2409	0.85	[0.83; 0.86]	2.1%	0.9%
Sjöström 2023	95	164	0.58	[0.50; 0.66]	0.3%	0.9%
Barman 2021	19	195	0.10	[0.06; 0.15]	0.1%	0.8%
See 2021	37	46	0.80	[0.66; 0.91]	0.0%	0.8%
Contreras-Villamizar 2023	474	690	0.69	[0.65; 0.72]	1.0%	0.9%
Bjornstad 2022	1926	4075	0.47	[0.46; 0.49]	7.0%	0.9%
Rahimzadeh 2021	55	79	0.70	[0.58; 0.79]	0.1%	0.8%
Bernardo 2022	87	120	0.72	[0.64; 0.80]	0.2%	0.8%
Fernández 2021	27	76	0.36	[0.25; 0.47]	0.1%	0.8%
Cheng 2020	74	140	0.53	[0.44; 0.61]	0.2%	0.9%
Kim 2023	2	40	0.05	[0.01; 0.17]	0.0%	0.6%
**Common effect model**		**67853**	**0.43**	**[0.43; 0.44]**	**100.0%**	.
**Random effects model**			**0.52**	**[0.48; 0.57]**	.	**100.0%**

Heterogeneity: $I^2 = 98\%$, $\tau^2 = 0.9421$, $p = 0$

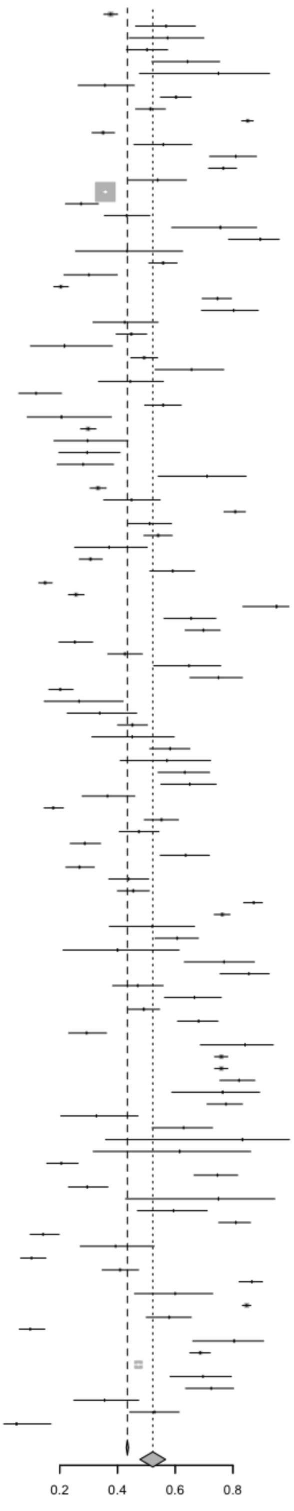

Figure S2. Forest Plot for ICU patients.

Subgroup	n_study	n_obs	n_events	Estimate
Overall	172	277943	63779	0.198 (0.173 to 0.226)
High income	98	214908	55143	0.260 (0.230 to 0.293)
United Kingdom	10	120629	31317	0.274 (0.232 to 0.321)
United States	37	60538	17614	0.303 (0.259 to 0.352)
Italy	12	8890	2054	0.255 (0.192 to 0.330)
Bahrain	1	50	29	0.580 (0.441 to 0.708)
Russian Federation	2	1570	433	0.276 (0.254 to 0.300)
Sweden	2	438	105	0.186 (0.064 to 0.433)
Portugal	3	675	361	0.512 (0.436 to 0.587)
Switzerland	1	188	41	0.218 (0.165 to 0.283)
Korea, Rep.	7	2873	476	0.149 (0.058 to 0.333)
Taiwan, China	2	266	47	0.089 (0.009 to 0.528)
Bulgaria	1	120	38	0.317 (0.240 to 0.405)
Spain	2	7597	822	0.108 (0.101 to 0.115)
Austria	1	130	39	0.300 (0.228 to 0.384)
Saudi Arabia	4	1693	519	0.291 (0.113 to 0.568)
France	3	842	189	0.327 (0.132 to 0.608)
Germany	1	85	14	0.165 (0.100 to 0.259)
Israel	2	2926	325	0.087 (0.044 to 0.164)
Canada	1	604	268	0.444 (0.405 to 0.484)
Poland	2	3894	372	0.093 (0.069 to 0.125)
Singapore	1	661	20	0.030 (0.020 to 0.046)
Japan	1	39	16	0.410 (0.269 to 0.568)
Hungary	1	141	28	0.199 (0.141 to 0.273)
Latvia	1	59	16	0.271 (0.173 to 0.398)
Upper middle income	51	43400	4103	0.110 (0.079 to 0.150)
Iran, Islamic Rep.	7	3794	983	0.253 (0.183 to 0.340)
China	23	30836	1366	0.049 (0.034 to 0.071)
Brazil	4	406	142	0.345 (0.208 to 0.513)
Türkiye	6	2094	208	0.069 (0.027 to 0.166)
Mexico	3	1267	513	0.450 (0.337 to 0.570)
Peru	1	349	38	0.109 (0.080 to 0.146)
Malaysia	1	241	11	0.046 (0.025 to 0.081)
Algeria	1	247	85	0.344 (0.288 to 0.406)
Colombia	3	1750	535	0.306 (0.283 to 0.330)
Iraq	1	2044	164	0.080 (0.069 to 0.093)
Argentina	1	372	58	0.156 (0.122 to 0.196)
Lower middle income	18	13299	2716	0.202 (0.132 to 0.296)
Pakistan	5	2827	988	0.296 (0.192 to 0.427)
Nepal	1	80	17	0.212 (0.136 to 0.316)
Nigeria	1	41	6	0.146 (0.067 to 0.290)
Egypt, Arab Rep.	3	496	123	0.315 (0.124 to 0.600)
India	5	6467	1216	0.135 (0.058 to 0.283)
Morocco	1	2354	40	0.017 (0.012 to 0.023)
Jordan	2	1034	326	0.301 (0.135 to 0.544)
Low income	3	562	79	0.141 (0.114 to 0.172)
Ethiopia	1	354	51	0.144 (0.111 to 0.185)
Congo, Dem. Rep.	1	106	15	0.142 (0.087 to 0.222)
Somalia	1	102	13	0.127 (0.075 to 0.207)
International	2	5774	1738	0.300 (0.268 to 0.335)

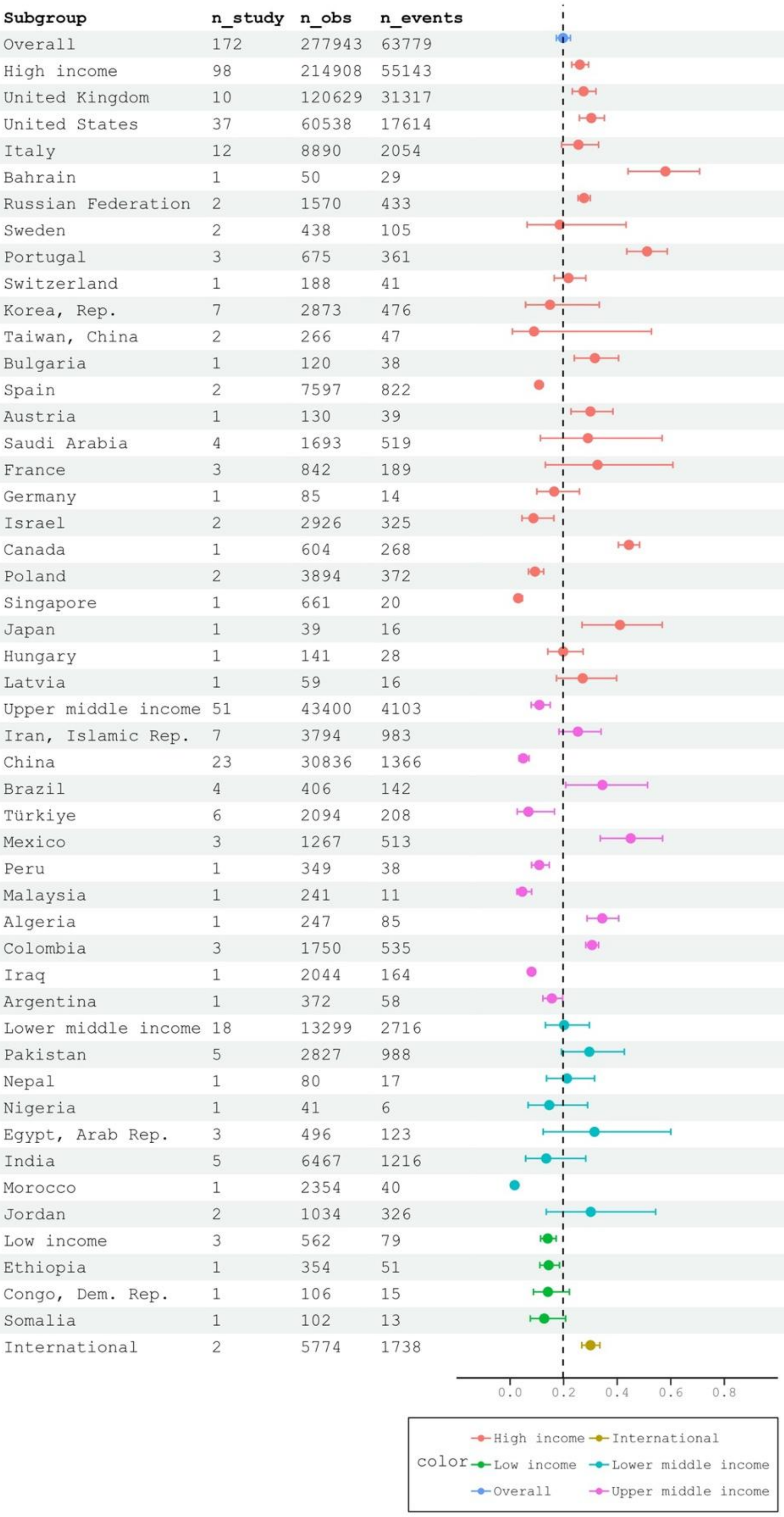

Figure S3. Non-ICU patients pooled AKI prevalence by countries coloured by income-level.

Subgroup	n_study	n_obs	n_events	Estimate
Overall	172	277943	63779	0.198 (0.173 to 0.226)
East Asia & Pacific	35	34916	1936	0.067 (0.045 to 0.097)
China	23	30836	1366	0.049 (0.034 to 0.071)
Korea, Rep.	7	2873	476	0.149 (0.058 to 0.333)
Taiwan, China	2	266	47	0.089 (0.009 to 0.528)
Malaysia	1	241	11	0.046 (0.025 to 0.081)
Singapore	1	661	20	0.030 (0.020 to 0.046)
Japan	1	39	16	0.410 (0.269 to 0.568)
Sub-Saharan Africa	4	603	85	0.141 (0.116 to 0.171)
Ethiopia	1	354	51	0.144 (0.111 to 0.185)
Nigeria	1	41	6	0.146 (0.067 to 0.290)
Congo, Dem. Rep.	1	106	15	0.142 (0.087 to 0.222)
Somalia	1	102	13	0.127 (0.075 to 0.207)
South Asia	11	9374	2221	0.204 (0.129 to 0.309)
Pakistan	5	2827	988	0.296 (0.192 to 0.427)
Nepal	1	80	17	0.212 (0.136 to 0.316)
India	5	6467	1216	0.135 (0.058 to 0.283)
Middle East & North Africa	22	14638	2594	0.228 (0.158 to 0.318)
Iran, Islamic Rep.	7	3794	983	0.253 (0.183 to 0.340)
Bahrain	1	50	29	0.580 (0.441 to 0.708)
Egypt, Arab Rep.	3	496	123	0.315 (0.124 to 0.600)
Saudi Arabia	4	1693	519	0.291 (0.113 to 0.568)
Algeria	1	247	85	0.344 (0.288 to 0.406)
Israel	2	2926	325	0.087 (0.044 to 0.164)
Morocco	1	2354	40	0.017 (0.012 to 0.023)
Jordan	2	1034	326	0.301 (0.135 to 0.544)
Iraq	1	2044	164	0.080 (0.069 to 0.093)
Europe & Central Asia	48	147352	36037	0.222 (0.183 to 0.268)
United Kingdom	10	120629	31317	0.274 (0.232 to 0.321)
Italy	12	8890	2054	0.255 (0.192 to 0.330)
Russian Federation	2	1570	433	0.276 (0.254 to 0.300)
Sweden	2	438	105	0.186 (0.064 to 0.433)
Türkiye	6	2094	208	0.069 (0.027 to 0.166)
Portugal	3	675	361	0.512 (0.436 to 0.587)
Switzerland	1	188	41	0.218 (0.165 to 0.283)
Bulgaria	1	120	38	0.317 (0.240 to 0.405)
Spain	2	7597	822	0.108 (0.101 to 0.115)
Austria	1	130	39	0.300 (0.228 to 0.384)
France	3	842	189	0.327 (0.132 to 0.608)
Germany	1	85	14	0.165 (0.100 to 0.259)
Poland	2	3894	372	0.093 (0.069 to 0.125)
Hungary	1	141	28	0.199 (0.141 to 0.273)
Latvia	1	59	16	0.271 (0.173 to 0.398)
North America	38	61142	17882	0.307 (0.263 to 0.355)
United States	37	60538	17614	0.303 (0.259 to 0.352)
Canada	1	604	268	0.444 (0.405 to 0.484)
Latin America & Caribbean	12	4144	1286	0.314 (0.234 to 0.406)
Brazil	4	406	142	0.345 (0.208 to 0.513)
Mexico	3	1267	513	0.450 (0.337 to 0.570)
Peru	1	349	38	0.109 (0.080 to 0.146)
Colombia	3	1750	535	0.306 (0.283 to 0.330)
Argentina	1	372	58	0.156 (0.122 to 0.196)
International	2	5774	1738	0.300 (0.268 to 0.335)

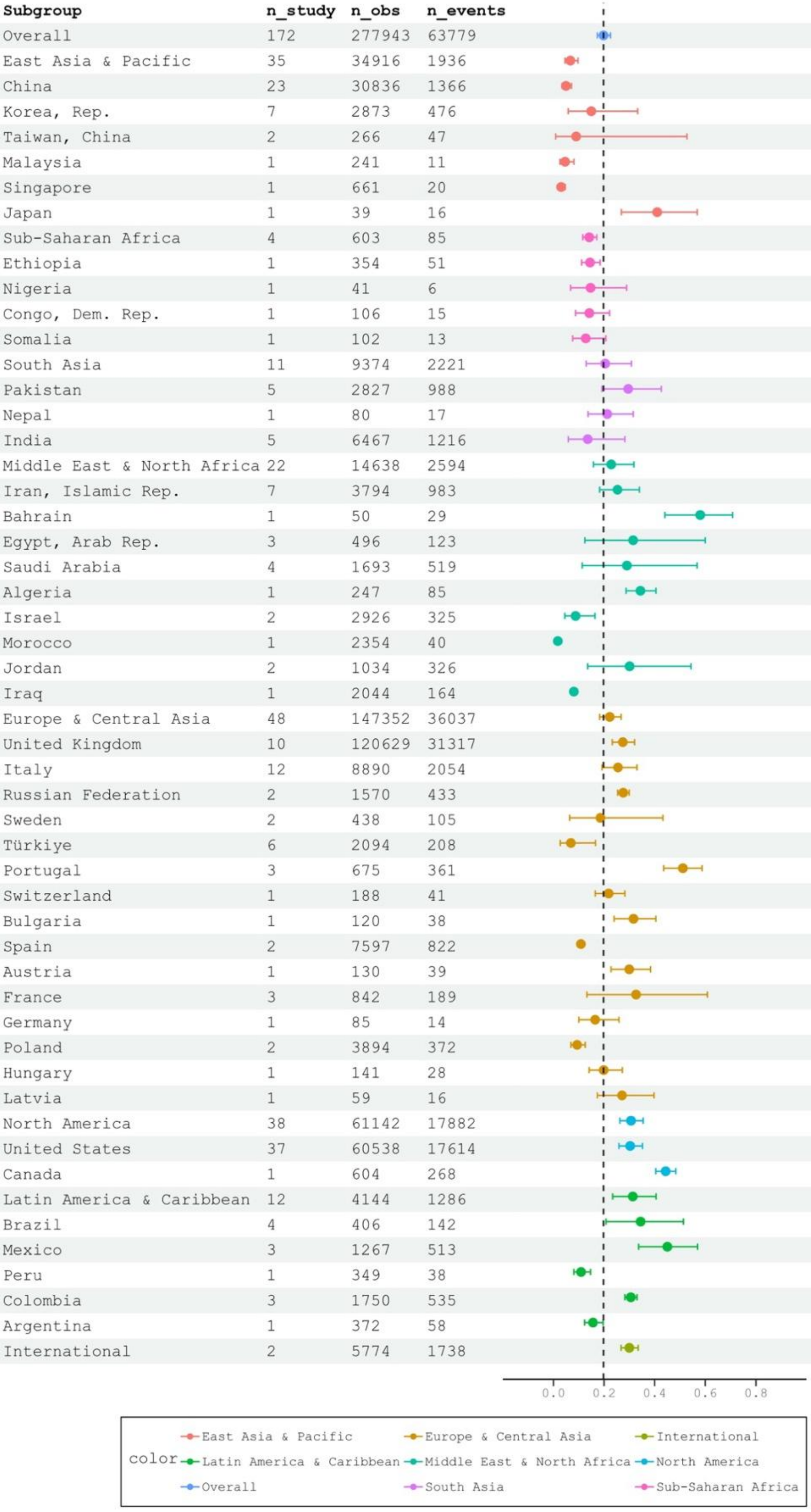

Figure S4. Non-ICU patients' pooled AKI prevalence by countries coloured by regions.

Subgroup	n_study	n_obs	n_events	Estimate
Overall	120	67853	30215	0.522 (0.478 to 0.567)
High income	63	20229	12322	0.602 (0.541 to 0.661)
Spain	2	1917	735	0.393 (0.349 to 0.440)
Italy	6	475	248	0.588 (0.350 to 0.790)
France	7	871	511	0.612 (0.464 to 0.741)
Saudi Arabia	2	395	238	0.603 (0.553 to 0.650)
Belgium	1	1286	1095	0.851 (0.831 to 0.870)
United Kingdom	9	2580	1089	0.500 (0.329 to 0.671)
Singapore	2	148	92	0.678 (0.382 to 0.878)
Germany	3	237	154	0.635 (0.372 to 0.836)
Sweden	4	375	236	0.663 (0.440 to 0.830)
United States	15	8836	6801	0.712 (0.629 to 0.783)
Austria	1	1042	267	0.256 (0.231 to 0.284)
Czechia	1	372	75	0.202 (0.164 to 0.245)
Latvia	1	530	94	0.177 (0.147 to 0.212)
Oman	1	286	158	0.552 (0.494 to 0.609)
Russian Federation	2	210	128	0.692 (0.259 to 0.935)
Portugal	2	158	119	0.770 (0.635 to 0.866)
Korea, Rep.	2	53	10	0.230 (0.010 to 0.895)
Canada	1	211	171	0.810 (0.752 to 0.858)
Poland	1	247	101	0.409 (0.349 to 0.471)
Upper middle income	40	7469	3238	0.477 (0.413 to 0.542)
Brazil	8	1961	903	0.592 (0.463 to 0.709)
Türkiye	10	1493	576	0.422 (0.278 to 0.580)
Argentina	2	238	97	0.405 (0.335 to 0.479)
China	8	840	329	0.391 (0.272 to 0.524)
Mexico	4	1340	442	0.493 (0.288 to 0.700)
Thailand	1	215	102	0.474 (0.409 to 0.541)
Iran, Islamic Rep.	5	686	310	0.487 (0.343 to 0.633)
Malaysia	1	6	5	0.833 (0.369 to 0.977)
Colombia	1	690	474	0.687 (0.651 to 0.720)
Lower middle income	9	2763	886	0.279 (0.194 to 0.384)
Morocco	2	806	231	0.201 (0.052 to 0.536)
India	3	1023	282	0.264 (0.209 to 0.327)
Tunisia	1	465	229	0.492 (0.447 to 0.538)
Egypt, Arab Rep.	2	293	54	0.201 (0.098 to 0.369)
Pakistan	1	176	90	0.511 (0.438 to 0.584)
Low income	1	48	25	0.521 (0.382 to 0.657)
Ethiopia	1	48	25	0.521 (0.382 to 0.657)
International	7	37344	13780	0.386 (0.272 to 0.513)

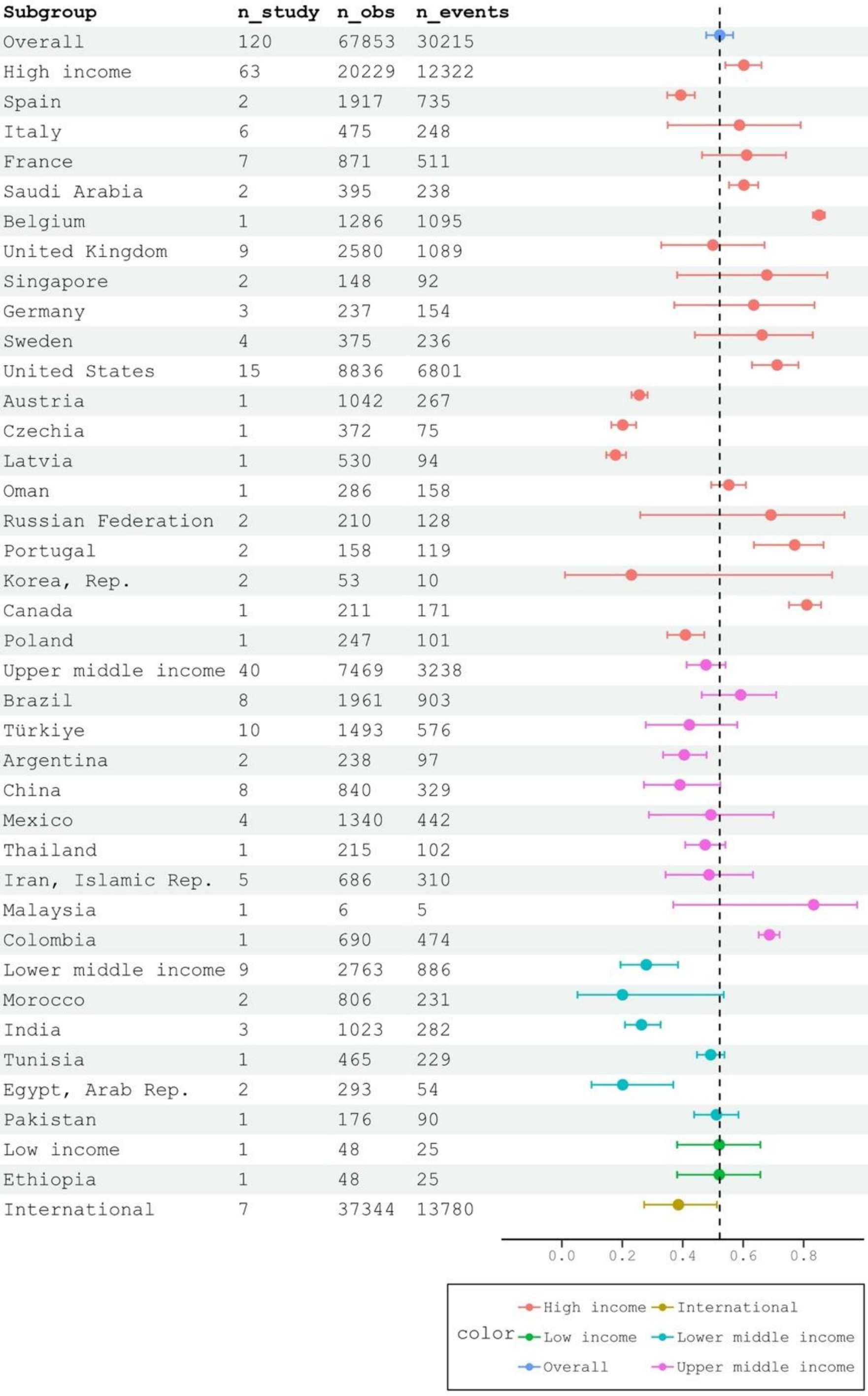

Figure S5. ICU patients' pooled AKI prevalence by countries coloured by income-level.

Subgroup	n_study	n_obs	n_events	Estimate
Overall	120	67853	30135	0.522 (0.478 to 0.567)
East Asia & Pacific	14	1262	538	0.441 (0.314 to 0.577)
Singapore	2	148	92	0.678 (0.382 to 0.878)
China	8	840	329	0.391 (0.272 to 0.524)
Thailand	1	215	102	0.474 (0.409 to 0.541)
Malaysia	1	6	5	0.833 (0.369 to 0.977)
Korea, Rep.	2	53	10	0.230 (0.010 to 0.895)
Sub-Saharan Africa	1	48	25	0.521 (0.382 to 0.657)
Ethiopia	1	48	25	0.521 (0.382 to 0.657)
South Asia	4	1199	372	0.315 (0.206 to 0.450)
India	3	1023	282	0.264 (0.209 to 0.327)
Pakistan	1	176	90	0.511 (0.438 to 0.584)
Middle East & North Africa	13	2931	1220	0.409 (0.299 to 0.530)
Saudi Arabia	2	395	238	0.603 (0.553 to 0.650)
Morocco	2	806	231	0.201 (0.052 to 0.536)
Tunisia	1	465	229	0.492 (0.447 to 0.538)
Egypt, Arab Rep.	2	293	54	0.201 (0.098 to 0.369)
Oman	1	286	158	0.552 (0.494 to 0.609)
Iran, Islamic Rep.	5	686	310	0.487 (0.343 to 0.633)
Europe & Central Asia	50	11793	5428	0.535 (0.461 to 0.606)
Spain	2	1917	735	0.393 (0.349 to 0.440)
Italy	6	475	248	0.588 (0.350 to 0.790)
France	7	871	511	0.612 (0.464 to 0.741)
Türkiye	10	1493	576	0.422 (0.278 to 0.580)
Belgium	1	1286	1095	0.851 (0.831 to 0.870)
United Kingdom	9	2580	1089	0.500 (0.329 to 0.671)
Germany	3	237	154	0.635 (0.372 to 0.836)
Sweden	4	375	236	0.663 (0.440 to 0.830)
Austria	1	1042	267	0.256 (0.231 to 0.284)
Czechia	1	372	75	0.202 (0.164 to 0.245)
Latvia	1	530	94	0.177 (0.147 to 0.212)
Russian Federation	2	210	128	0.692 (0.259 to 0.935)
Portugal	2	158	119	0.770 (0.635 to 0.866)
Poland	1	247	101	0.409 (0.349 to 0.471)
North America	16	9047	6972	0.719 (0.641 to 0.786)
United States	15	8836	6801	0.712 (0.629 to 0.783)
Canada	1	211	171	0.810 (0.752 to 0.858)
Latin America & Caribbean	15	4229	1916	0.546 (0.451 to 0.638)
Brazil	8	1961	903	0.592 (0.463 to 0.709)
Argentina	2	238	97	0.405 (0.335 to 0.479)
Mexico	4	1340	442	0.493 (0.288 to 0.700)
Colombia	1	690	474	0.687 (0.651 to 0.720)
International	7	37344	13780	0.386 (0.272 to 0.513)

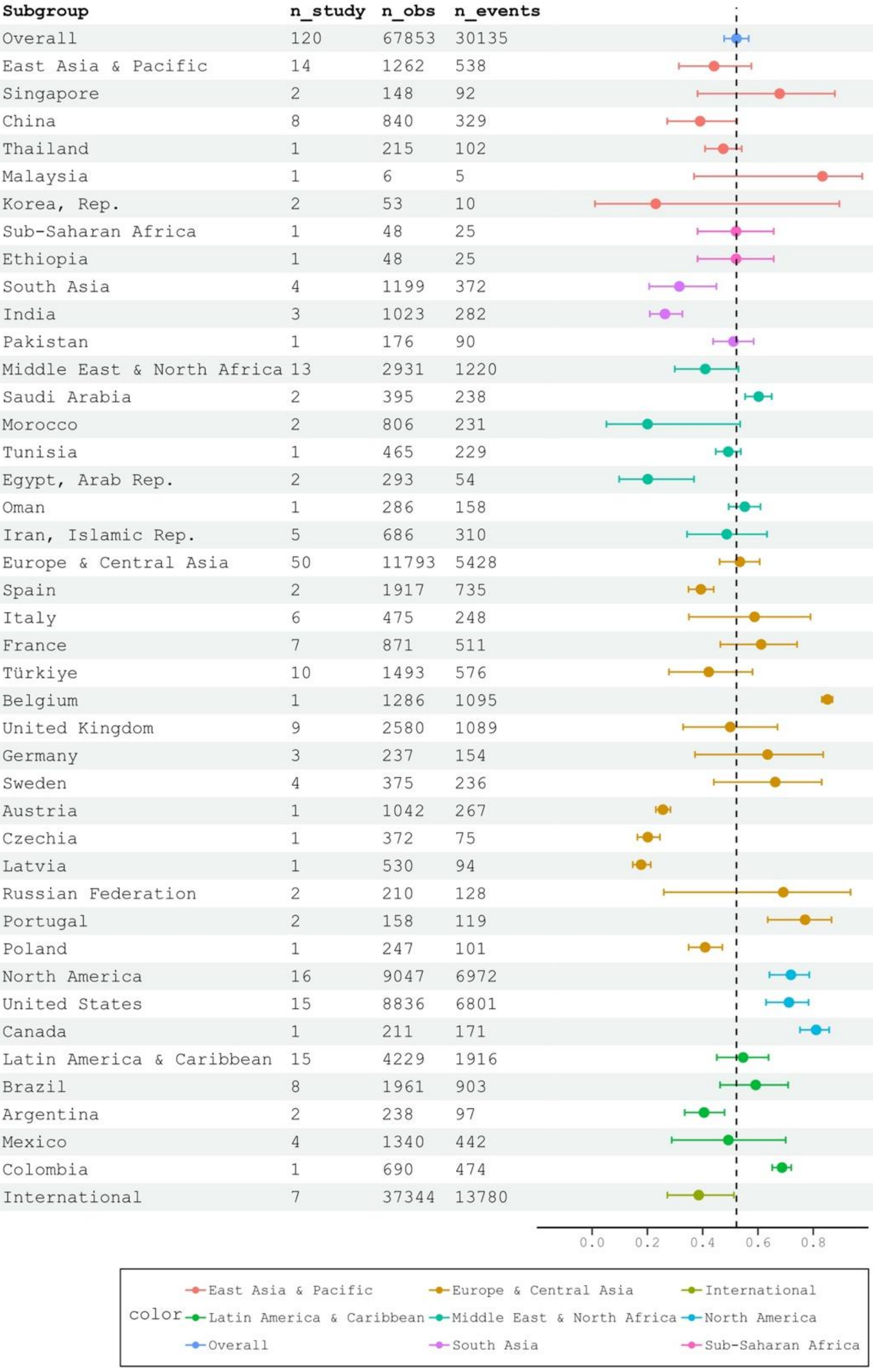

Figure S6. ICU patient's pooled AKI prevalence by countries coloured by regions.

Searching Strategies

PubMed:

Final research design: Published date: 01/12/2019 to 31/12/2023, English Only, Species Human, adult only studies NO Casereport– searched on 14/11/2023: 678

(("2019 ncov"[Title/Abstract] OR "SARS-CoV-2"[MeSH Terms] OR "COVID-19"[MeSH Terms] OR "coronavirus disease 2019"[Title/Abstract] OR "novel coronavirus"[Title/Abstract] OR "2019 ncov"[Title/Abstract] OR "novel coronavirus 2019"[Title/Abstract]) AND ("acute kidney injury"[MeSH Terms] OR "acute kidney failure"[Title/Abstract] OR "acute renal failure"[Title/Abstract] OR "acute renal injury"[Title/Abstract] OR "AKI"[Title/Abstract] OR "kidney injury"[Title/Abstract] OR "renal impairment"[Title/Abstract] OR "acute renal insufficiencies"[Title/Abstract] OR "acute kidney insufficiencies"[Title/Abstract] OR "acute kidney tubular necrosis"[Title/Abstract]) AND (("humans"[MeSH Terms] AND "english"[Language] AND ("adult"[MeSH Terms] OR "adult"[MeSH Terms:noexp] OR ("middle aged"[MeSH Terms] OR "aged"[MeSH Terms]) OR "middle aged"[MeSH Terms] OR "aged"[MeSH Terms] OR "aged, 80 and over"[MeSH Terms] OR "young adult"[MeSH Terms]) AND 2019/01/01:2023/12/31[Date - Publication]) NOT "case reports"[Publication Type])) AND ((humans[Filter]) AND (english[Filter]) AND (alladult[Filter] OR youngadult[Filter] OR adult[Filter] OR middleagedaged[Filter] OR middleaged[Filter] OR aged[Filter] OR 80andover[Filter]))

Embase:

Final search design: Published date: 2020 to 2023, English Only, Species Human, journal article, editorial, chapter, Adult, young adult, middle aged, aged, very elderly, NOT (case reports) searched on 20/11/2023: 2127

('sars cov 2'/exp OR 'covid 19'/exp OR 'coronavirus disease 2019':ti,ab OR 'novel coronavirus':ti,ab OR '2019 ncov':ti,ab OR 'novel coronavirus 2019':ti,ab) AND ('acute kidney injury'/exp OR 'acute kidney failure':ti,ab OR 'acute renal failure':ti,ab OR 'acute renal injury':ti,ab OR aki:ti,ab OR 'kidney injury':ti,ab OR 'renal impairment':ti,ab OR 'acute renal insufficiencies':ti,ab OR 'acute kidney insufficiencies':ti,ab OR 'acute kidney tubular necrosis':ti,ab) AND [humans]/lim AND [english]/lim AND [2019-2023]/py AND ([adult]/lim OR [aged]/lim OR [middle aged]/lim OR [very elderly]/lim OR [young adult]/lim) AND ('article'/it OR 'chapter'/it OR 'editorial'/it OR 'letter'/it) NOT (('sars cov 2'/exp OR 'covid 19'/exp OR 'coronavirus disease 2019':ti,ab OR 'novel coronavirus':ti,ab OR '2019 ncov':ti,ab OR 'novel coronavirus 2019':ti,ab) AND ('acute kidney injury'/exp OR 'acute kidney failure':ti,ab OR 'acute renal failure':ti,ab OR 'acute renal injury':ti,ab OR aki:ti,ab OR 'kidney injury':ti,ab OR 'renal impairment':ti,ab OR 'acute renal insufficiencies':ti,ab OR 'acute kidney insufficiencies':ti,ab OR 'acute kidney tubular

necrosis':ti,ab) AND [humans]/lim AND [english]/lim AND [2019-2023]/py AND ([adult]/lim OR [aged]/lim OR [middle aged]/lim OR [very elderly]/lim OR [young adult]/lim) AND ('article'/it OR 'chapter'/it OR 'editorial'/it OR 'letter'/it) AND ('case report'/de OR 'case study'/de))

Scopus:

Final search design: Published date: 2020 to 2023, English Only, Adult only, excluding case reports, letter, note, short survey, searched on 20/11/2023: 1330

(TITLE-ABS (2019-ncov) OR INDEXTERMS (sars-cov-2) OR INDEXTERMS (covid-19) OR TITLE-ABS ("coronavirus disease 2019") OR TITLE-ABS ("novel coronavirus") OR TITLE-ABS ("2019 nCov") OR TITLE-ABS ("novel coronavirus 2019") OR INDEXTERMS ("Coronavirus Disease 2019") OR INDEXTERMS ("Severe Acute Respiratory Syndrome Coronavirus 2")) AND (INDEXTERMS ("Acute Kidney Injury") OR INDEXTERMS ("Acute Kidney Failure ") OR TITLE-ABS ("acute kidney failure") OR TITLE-ABS ("acute renal failure") OR TITLE-ABS ("acute renal injury") OR TITLE-ABS (aki) OR TITLE-ABS ("kidney injury") OR TITLE-ABS ("renal impairment") OR TITLE-ABS ("acute renal insufficiencies") OR TITLE-ABS ("acute kidney insufficiencies") OR TITLE-ABS ("acute kidney tubular necrosis")) AND PUBYEAR > 2018 AND PUBYEAR < 2024 AND (EXCLUDE (DOCTYPE , "cp")) AND (LIMIT-TO (LANGUAGE , "English")) AND (LIMIT-TO (EXACTKEYWORD , "Child") OR LIMIT-TO (EXACTKEYWORD , "Case Report"))

Web of Science:

((TI=2019-nCoV OR AB=2019-nCoV) OR ALL=SARS-CoV-2 OR ALL=COVID-19 OR (TI="coronavirus disease 2019" OR AB="coronavirus disease 2019") OR (TI="novel coronavirus" OR AB="novel coronavirus") OR (TI="2019 nCov" OR AB="2019 nCov") OR (TI="novel coronavirus 2019" OR AB="novel coronavirus 2019")) AND (ALL="acute kidney injury" OR (TI="acute kidney failure" OR AB="acute kidney failure") OR (TI="acute renal failure" OR AB="acute renal failure") OR (TI="acute renal injury" OR AB="acute renal injury") OR (TI=AKI OR AB=AKI) OR (TI="kidney injury" OR AB="kidney injury") OR (TI="renal impairment" OR AB="renal impairment") OR (TI="acute renal insufficiencies" OR AB="acute renal insufficiencies") OR (TI="acute kidney insufficiencies" OR AB="acute kidney insufficiencies") OR (TI="acute kidney tubular necrosis" OR AB="acute kidney tubular necrosis")) NOT (TI="kidney transplant")

Cochrane:

[mh SARS-CoV-2] OR [mh COVID-19] OR "coronavirus disease 2019":ti,ab,kw OR "novel coronavirus":ti,ab,kw OR "2019 nCov":ti,ab,kw OR "novel coronavirus 2019":ti,ab,kw) AND ([mh "acute kidney injury"] OR "acute kidney failure":ti,ab,kw OR "acute renal

failure":ti,ab,kw OR "acute renal injury":ti,ab,kw OR AKI:ti,ab,kw OR "kidney injury":ti,ab,kw OR "renal impairment":ti,ab,kw OR "acute renal insufficiencies":ti,ab,kw OR "acute kidney insufficiencies":ti,ab,kw OR "acute kidney tubular necrosis":ti,ab,kw

Funnel Plots for bias

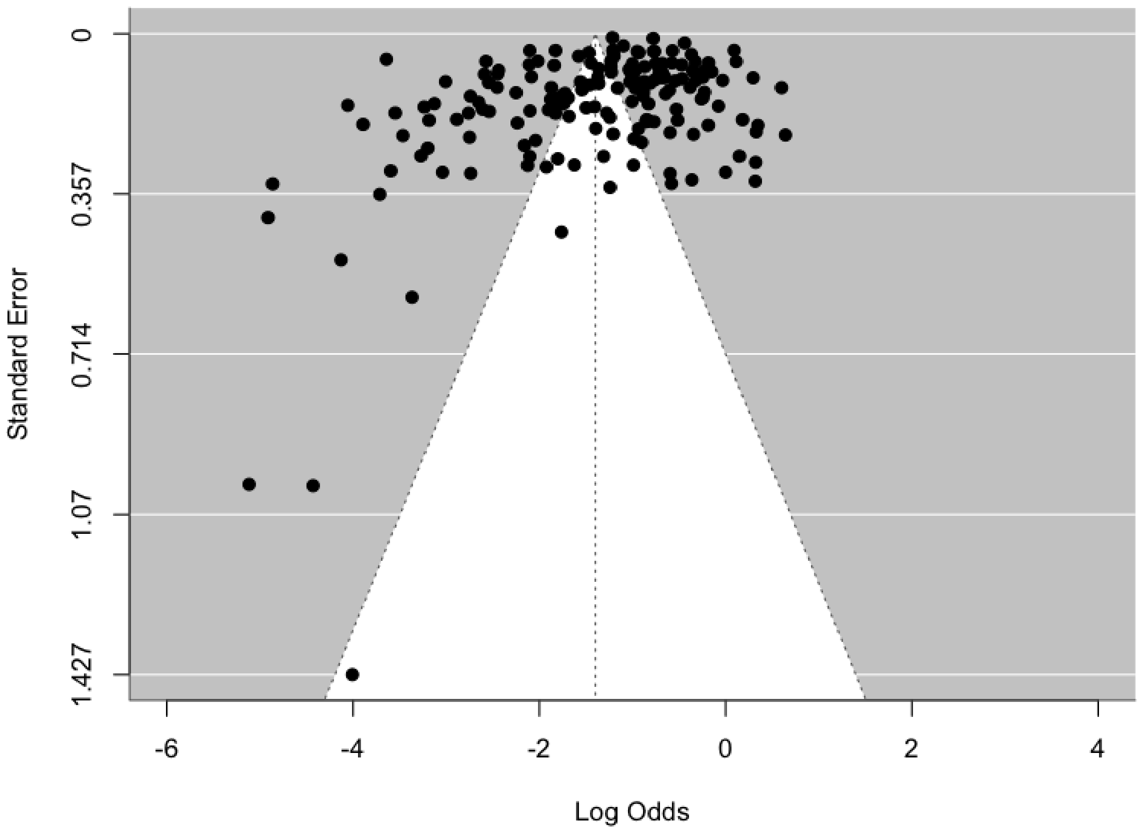

Figure S7. Non-ICU AKI prevalence funnel plot.

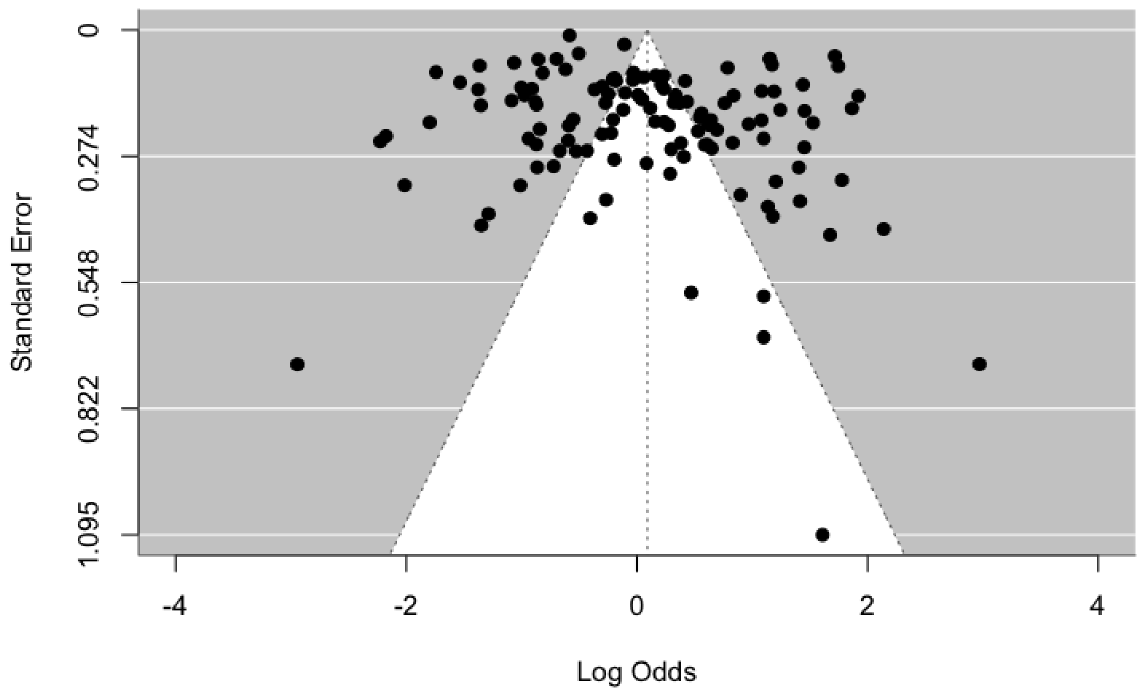

Figure S8. ICU AKI prevalence funnel plot.
